# Genomic Instability, Defective Spermatogenesis, Immunodeficiency, and Cancer in a Mouse Model of the RIDDLE Syndrome

**DOI:** 10.1371/journal.pgen.1001381

**Published:** 2011-04-28

**Authors:** Toshiyuki Bohgaki, Miyuki Bohgaki, Renato Cardoso, Stephanie Panier, Dimphy Zeegers, Li Li, Grant S. Stewart, Otto Sanchez, M. Prakash Hande, Daniel Durocher, Anne Hakem, Razqallah Hakem

**Affiliations:** 1 Ontario Cancer Institute, University Health Network and Department of Medical Biophysics, University of Toronto, Toronto, Canada; 2 Samuel Lunenfeld Research Institute, Mount Sinai Hospital, Toronto, Canada; 3 Department of Physiology, Yong Loo Lin School of Medicine, National University of Singapore, Singapore, Singapore; 4 Cancer Research UK, Institute for Cancer Studies, Birmingham University, Birmingham, United Kingdom; 5 University of Ontario Institute of Technology, Oshawa, Canada; National Cancer Institute, United States of America

## Abstract

Eukaryotic cells have evolved to use complex pathways for DNA damage signaling and repair to maintain genomic integrity. RNF168 is a novel E3 ligase that functions downstream of ATM,γ-H2A.X, MDC1, and RNF8. It has been shown to ubiquitylate histone H2A and to facilitate the recruitment of other DNA damage response proteins, including 53BP1, to sites of DNA break. In addition, RNF168 mutations have been causally linked to the human RIDDLE syndrome. In this study, we report that *Rnf168^−/−^* mice are immunodeficient and exhibit increased radiosensitivity. *Rnf168^−/−^* males suffer from impaired spermatogenesis in an age-dependent manner. Interestingly, in contrast to *H2a.x^−/−^*, *Mdc1^−/−^*, and *Rnf8^−/−^* cells, transient recruitment of 53bp1 to DNA double-strand breaks was abolished in *Rnf168^−/−^* cells. Remarkably, similar to 53bp1 inactivation, but different from H2a.x deficiency, inactivation of Rnf168 impairs long-range V(D)J recombination in thymocytes and results in long insertions at the class-switch junctions of B-cells. Loss of Rnf168 increases genomic instability and synergizes with p53 inactivation in promoting tumorigenesis. Our data reveal the important physiological functions of Rnf168 and support its role in both γ-H2a.x-Mdc1-Rnf8-dependent and -independent signaling pathways of DNA double-strand breaks. These results highlight a central role for RNF168 in the hierarchical network of DNA break signaling that maintains genomic integrity and suppresses cancer development in mammals.

## Author Summary

The repair of DNA damage is fundamental as illustrated by the many human syndromes, immunodeficiencies, and cancers associated with defects in DNA damage signaling and repair. RIDDLE syndrome, caused by mutations of the human *RNF168*, is a novel hereditary disease clinically characterized by radiosensitivity, immunodeficiency, dysmorphic features, and learning difficulties. RNF168 is an E3 ligase that modifies histones and chromatin structure at sites of DNA breaks. In this study, we show that Rnf168 deficiency in mice leads to increased radiosensitivity, immunodeficiency, and defective spermatogenesis. Additionally, dual inactivation of Rnf168 and p53 leads to increased cancer risk. Collectively these data demonstrate important and broad physiological functions for Rnf168.

## Introduction

DNA damage checkpoint signaling and DNA repair pathways are key elements of the DNA damage response (DDR) and are critical for the maintenance of genomic integrity [1]–[3]. Mammalian cells constantly experience DNA damage as a result of exogenous exposure to ionizing radiation (IR), ultraviolet light (UV), chemical compounds, and radical oxygen species, as well as endogenous insults due to DNA replication errors. In addition, double-strand DNA breaks (DSBs) are also programmed to occur during immune-receptor rearrangements and meiosis.

Mutations of genes involved in DNA damage signaling or repair can lead to many diseases including neurodegenerative diseases, immunodeficiency and cancer, underlining the importance of these processes [1], [4], [5]. Among the various types of DNA damage, DSBs are the most serious and require elaborated networks of proteins to signal and repair the damage. In mammalian cells, DSBs are initially recognized by the Mre11/Rad50/NBS1 (MRN) complex that induces the activation and recruitment of the ataxia-telangiectasia-mutated (ATM) kinase to the break sites [6]–[8]. At the flanking sites of DSBs, H2A.X, a variant of the histone H2A, is rapidly phosphorylated at the serine 139 residue (γ-H2A.X). Phosphorylation of H2A.X is mediated by activated ATM, which itself is phosphorylated at serine 1981 (phospho-ATM-S1981), or alternatively by two other phosphoinositide 3-kinase like kinases (PIKKs), namely the ataxia telangiectasia and Rad3 related (ATR) and the DNA-dependent protein kinase catalytic subunit (DNA-PKcs). Active ATM also phosphorylates a number of other proteins including the structural maintenance of chromosomes 1 (SMC1), the Nijmegen breakage syndrome protein 1 (NBS1), the checkpoint kinase 2 (Chk2), the breast cancer 1- early onset (BRCA1) and the mediator of DNA damage checkpoint protein-1 (MDC1). Subsequent to its phosphorylation, MDC1 binds to γ-H2A.X via its tandem BRCA1 C-terminal (BRCT) domains, and recruits additional active ATM to sites flanking the DSBs [7], [9], [10]. MDC1 also associates with MRN complex through its Ser-Asp-Thr (SDT) repeats and the fork-head associated (FHA) domain of NBS1. Furthermore, through its C-terminus, NBS1 also interacts with ATM [11]–[15]. As such, MDC1 bridges the interaction of MRN to γ-H2A.X and ATM. Enrichment of ATM-MDC1 and ATM-MRN at the break sites further amplify the phosphorylation of H2A.X and triggers the recruitment of other DNA damage response proteins to the DSB flanking regions [16]. The three conserved T-Q-X-F clusters between 698 to 800 amino acids of MDC1 are also phosphorylated by ATM. The threonine-phosphorylated MDC1 has been shown to interact with the FHA domain of RING finger protein 8 (RNF8), thus recruiting this E3 ligase to the sites of damage. Subsequently, RNF8, along with the E2 ubiquitin-conjugating enzyme UBC13, mediate lysine 63 (K63)-linked ubiquitylation of the histone H2A at the flanking regions of DSBs [17]–[19]. Such ubiquitylated form of H2A interacts with the MIU2 (motif interacting with ubiquitin 2) domain of RNF168 and recruits this E3 ligase to DSB sites, allowing it to further ubiquitylate surrounding H2A [20]–[22]. This likely amplifies the modification of chromatin structure at regions adjacent to DSBs, and facilitates the recruitment of tumor suppressor p53 binding protein 1 (53BP1). In addition, RAP80 selectively binds to the K63-polyubiquitin chains on H2A via its tandem ubiquitin interaction motifs (UIMs) [23], and acts as a bridge to recruit BRCA1 to the regions of DSBs. In sum, DSBs signaling is a highly regulated process, in which RNF168 plays a major role through its contribution to the recruitment of various downstream DDR proteins.

*RNF168* is mutated in RIDDLE syndrome, which is characterized by radiosensitivity, immunodeficiency, dysmorphic features and learning difficulties [24]. RNF168 contains a RING finger domain and two MIU domains. The RING finger domain of RNF168 is critical for its ubiquitin E3 ligase activity, whereas its MIU2 domain mediates its binding to ubiquitylated H2A [20]–[22]. Knockdown of RNF168 in human cells significantly impaired the formation of 53BP1, RAP80 and BRCA1 ionizing radiation induced foci (IRIF). While recent studies have revealed a role for RNF168 in ubiquitylating H2A during DSB signaling [20]–[22], the full physiological functions of RNF168 are not understood.

Here, we report the generation of a mouse model for RNF168 deficiency. *Rnf168^−/−^* mice are viable. Consistent with the clinical features of RIDDLE syndrome, *Rnf168^−/−^* mice are immunodeficient, and their cells show increased radiosensitivity. Similar to 53bp1 deficiency, long-range V(D)J recombination of T-cell receptor (TCR)δ is also impaired in *Rnf168^−/−^* thymocytes, while aberrant long insertions have been observed at class switch junctions of *Rnf168^−/−^* B-lymphocytes. Moreover, in contrast to the transient recruitment of 53bp1 to DSBs in H2a.x-, Mdc1- and Rnf8-deficient mouse embryonic fibroblasts (MEFs), 53bp1 recruitment to DSB sites is completely abolished in Rnf168-deficient MEFs. Our data also demonstrate novel roles of Rnf168 in spermatogenesis, maintenance of genomic integrity and cancer, and therefore, further highlight the other important physiological functions of this molecule.

## Results

### Rnf168 is dispensable for embryonic and postnatal development

To investigate the physiological role of Rnf168, we generated Rnf168 deficient mice using two different gene trap embryonic stem cell (ES) clones (156B6 and 405F11). Gene trap of *Rnf168* in the two ES clones was confirmed by Southern blot, PCR and sequencing of the genomic site of the retrovirus integration (Figure S1A and S1B). RT-PCR revealed the disruption of full length *Rnf168* transcript in the two *Rnf168* gene trap ES clones (Figure S1C). The loss of Rnf168 protein was also confirmed in *Rnf168^−/−^* MEFs and splenocytes by immunoblotting (Figure S1D and S1E).

Intercrossing of *Rnf168^+/−^* mice obtained with 156B6 and 405F11 ES clones indicated that *Rnf168^−/−^* mice were viable and born at the expected Mendelian ratio (Table S1). *Rnf168^−/−^* mice did not exhibit any gross developmental defects and were indistinguishable from their *wildtype* (*WT*) littermates. Collectively, these data indicate that the E3 ligase Rnf168 is not required for embryonic and postnatal development.

### Rnf168 deficiency leads to increased radiation sensitivity

**Figure 1:**
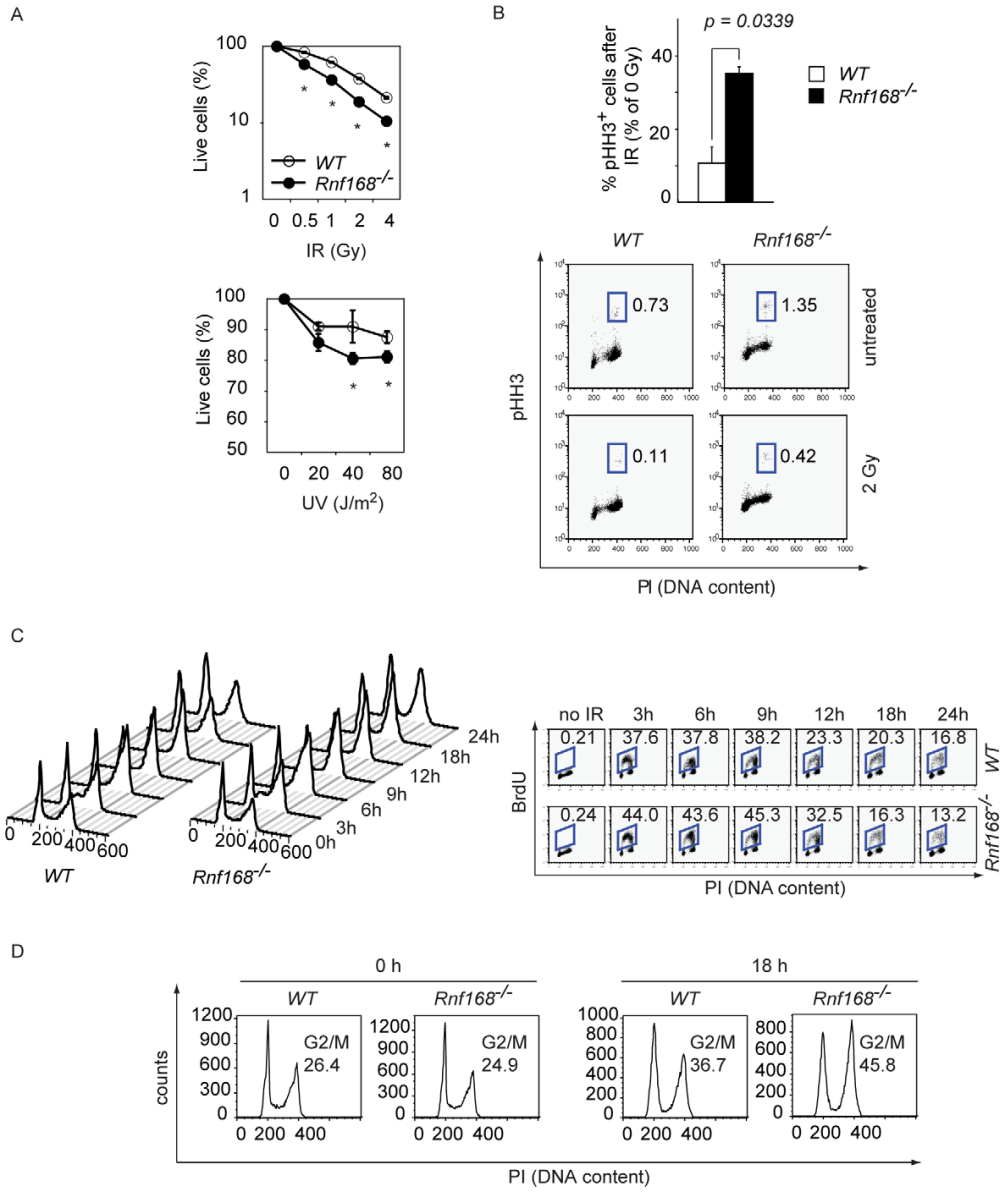
Impaired DNA damage response in Rnf168 deficient cells. (A) Cells were subjected to IR or UV, and the extent of cell death was determined 24 hours later. Three independent experiments were performed. Data are presented as the mean ± SEM. *p<0.05. (B) Primary MEFs (passage 2) either untreated or irradiated (2 Gy) were stained with anti-phospho-histone H3 (pHH3) antibody and PI at 1 hour post IR. The percentage of mitotic cells was determined by FACS. Three independent experiments were performed. Data are presented as the mean ± SEM. *p<0.05. (C) Cell cycle progression post IR (10 Gy) treatment of *WT* and *Rnf168^−/−^* primary MEFs (Passage 2) was examined using BrdU/PI assay and FACS. Representative data are shown for three independent experiments. (D) Accumulation at the G2 phase of *WT* and *Rnf168^−/−^* primary MEFs (passage 2) post IR (10 Gy). Cell cycle profiles were examined by PI staining and representative data are shown from three independent experiments.

Mutation of the *RNF168* gene is associated with RIDDLE syndrome [22], [24]. To determine whether Rnf168 deficiency in mice confers increased sensitivity to DNA damage, primary lymphocytes isolated from peripheral lymph nodes (LN) of *Rnf168^−/−^* mice and *WT* littermates were subjected to various doses of IR or UV. Lymphocyte viability was examined using 7-aminoactinomycin D (7AAD) and flow cytometry. These studies demonstrate a significant increase in the sensitivity of *Rnf168^−/−^* lymphocytes to IR and UV treatment (Figure 1A).

DNA damage signaling plays essential roles in the activation of checkpoints and cell cycle progression [25]. Examination of the effect of Rnf168 inactivation on cell cycle progression using G1/S synchronized MEFs showed no difference between *Rnf168^−/−^* and *WT* MEFs (Figure S2A).

*Atm^−/−^*, *H2a.x^−/−^*, *Mdc1^−/−^*, *Rnf8* ^−/−^ and *53bp1^−/−^* cells exhibit a defect in the early G2/M checkpoint [10], [17]–[19], [26]–[28]. Atm and 53bp1-deficient cells display late G2/M accumulation and inactivation of 53bp1 results in a mild impairment of the intra-S phase checkpoint [26], [28]. Therefore, we examined the effects of Rnf168 deficiency on cell cycle checkpoints. Immunostaining of irradiated *Rnf168^−/−^* and *WT* MEFs with anti-phospho-histone H3 (pHH3), and co-staining with propidium iodide (PI) demonstrated a higher proportion of irradiated *Rnf168^−/−^* MEFs (35.2±1.8%) have progressed to M phase compared to *WT* MEFs (10.7±4.3%, *p<0.05*) (Figure 1B). Next, the effect of irradiation on cell cycle progression of *Rnf168^−/−^* MEFs and *WT* controls was examined using the Bromodeoxiuridine (BrdU) and PI assay. The percentage of *Rnf168^−/−^* cells in S phase 3 hours post-IR (10 Gy) was slightly increased compared to *WT* cells (Figure 1C). These data suggest that similar to *53bp1^−/−^* MEFs [24], [28], *Rnf168^−/−^* MEFs also have a mild defect in the intra-S phase checkpoint. In addition, cell cycle analyses of *Rnf168^−/−^* and *WT* MEFs 18 hours post-IR indicated that a higher proportion of the *Rnf168^−/−^* MEFs is found at the G2/M phase (Figure 1D). Therefore, similar to H2a.x, Mdc1, Rnf8 and 53bp1, Rnf168 is dispensable for the activation of the G1/S checkpoint, but is important for enforcing the G2/M DNA damage checkpoint.

### Rnf168 promotes the recruitment of 53bp1 and Brca1 to DNA breaks

**Figure 2:**
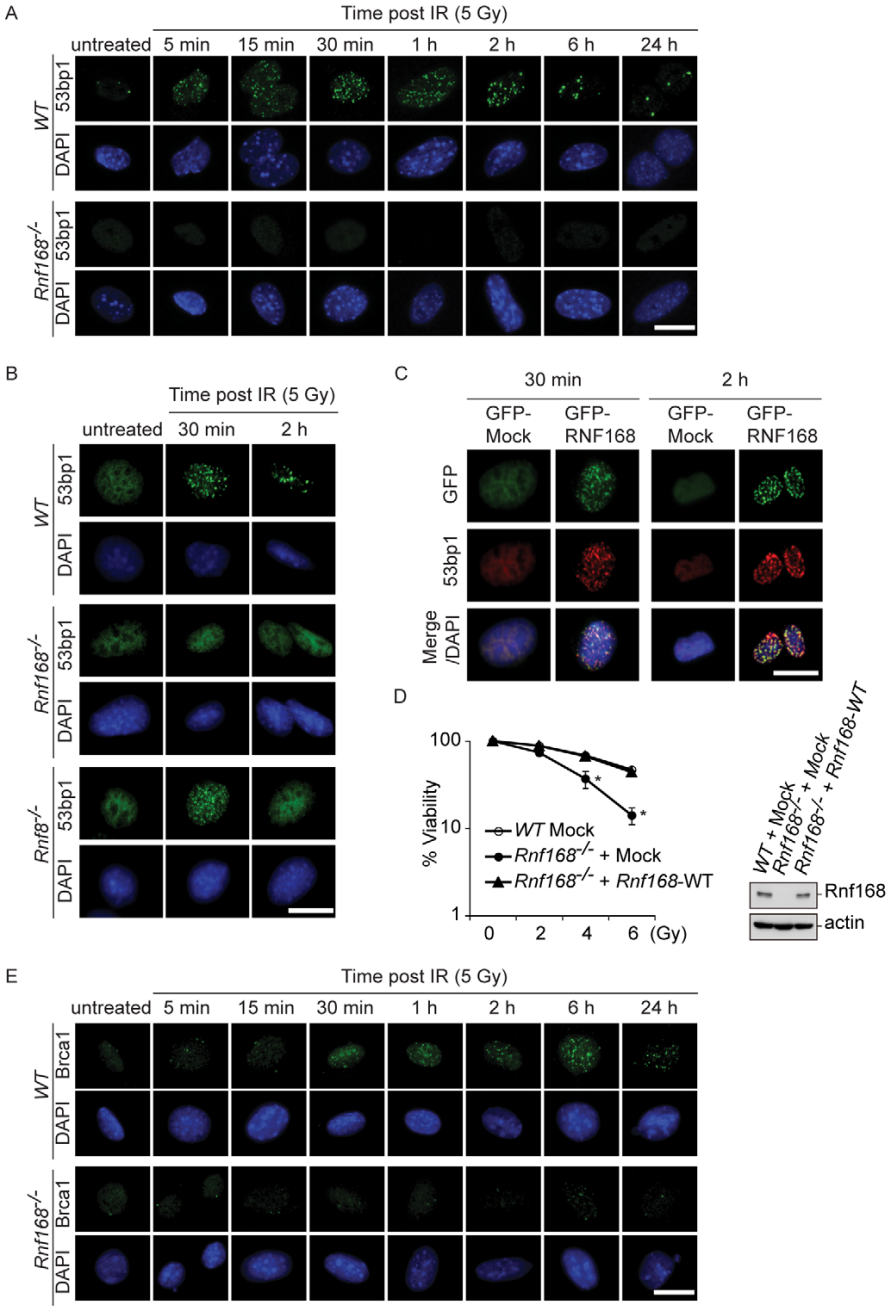
Rnf168 is required for the recruitment of 53bp1 to DNA damage sites. (A) Representative micrographs of MEFs stained with anti-53bp1 antibody and DAPI. *Rnf168^−/−^* and *WT* MEFs were untreated or exposed to 5 Gy of IR and fixed at the indicated time post IR. Bars, 20 µm. (B) Representative micrographs of MEFs stained with anti-53bp1 antibody and DAPI are shown. *Rnf168^−/−^*, *Rnf8^−/−^* and *WT* MEFs were untreated or exposed to 5 Gy of IR and fixed at the indicated time post IR. Three independent experiments were performed. Bars, 20 µm. (C) Recovery of 53bp1 IRIF formation in *Rnf168^−/−^* MEFs complemented with full length RNF168. Immortalized *Rnf168^−/−^* MEFs were mock-transfected or transfected with GFP-tagged RNF168 expression vectors and cultured for 24 hours. Cells were fixed at 1 hour post-IR (5 Gy) and processed for immunofluorescence staining with anti-53bp1 antibodies. Three independent experiments were performed. Bars, 20 µm. (D) Clonogenic assay was performed to examine radiosensitivity of *Rnf168^−/−^* MEFs complemented with exogenous Rnf168 (left panel). Expression level of Rnf168 is shown for the MEFs used for the clonogenic assay (right panel). Data shown is representative of three independent experiments and is presented as the mean ± SEM. *p<0.05. (E) Representative micrographs of MEFs stained with anti-Brca1 antibody and DAPI. *Rnf168^−/−^* and *WT* MEFs were either untreated or exposed to 5 Gy of IR and fixed at the indicated time after IR. Bars, 2 µm.

Through ubiquitylation of histone H2A, RNF168 plays an important role in the recruitment of 53BP1 to the sites of DNA breaks downstream of H2A.X, MDC1 and RNF8 [20]–[22]. Although H2a.x, Mdc1 and Rnf8 are each essential for the stable ‘retention’ of 53bp1 foci at DSB sites, studies of *H2a.x^−/−^*, *Mdc1^−/−^* and *Rnf8^−/−^* MEFs demonstrate that these cells can still transiently recruit 53bp1 to form IRIF [29]. In addition, a partial accumulation of 53bp1 at DSBs has been also observed in activated *Rnf8^−/−^* B-cells post-irradiation [30]. To investigate the effect of Rnf168 deficiency on the recruitment of 53bp1 to sites of DSBs, *Rnf168^−/−^*, *Rnf8^−/−^* and *WT* MEFs were subjected to 5 Gy of IR, followed by a time course analysis of 53bp1 foci formation by immunofluorescence microscopy. In contrast to *H2a.x^−/−^*, *Mdc1^−/−^* and *Rnf8^−/−^* MEFs [5], no 53bp1 IRIF were observed in *Rnf168^−/−^* MEFs post-IR (Figure 2A and 2B and Figure S3A). Transfection of *Rnf168^−/−^* MEFs with GFP-tagged RNF168 expression constructs fully restored formation of 53bp1 IRIF (Figure 2C). In addition, radiosensitivity of *Rnf168^−/−^* MEFs was also rescued by expression of Rnf168 (Figure 2D). *Rnf168* deficiency also significantly impaired Brca1 recruitment to sites of DSBs (Figure 2E and Figure S3B). These data indicate an important role for Rnf168 in the recruitment of both 53bp1 and Brca1 to the DNA break sites.

### Rnf168 is dispensable for the activation of Atm signaling pathway

**Figure 3:**
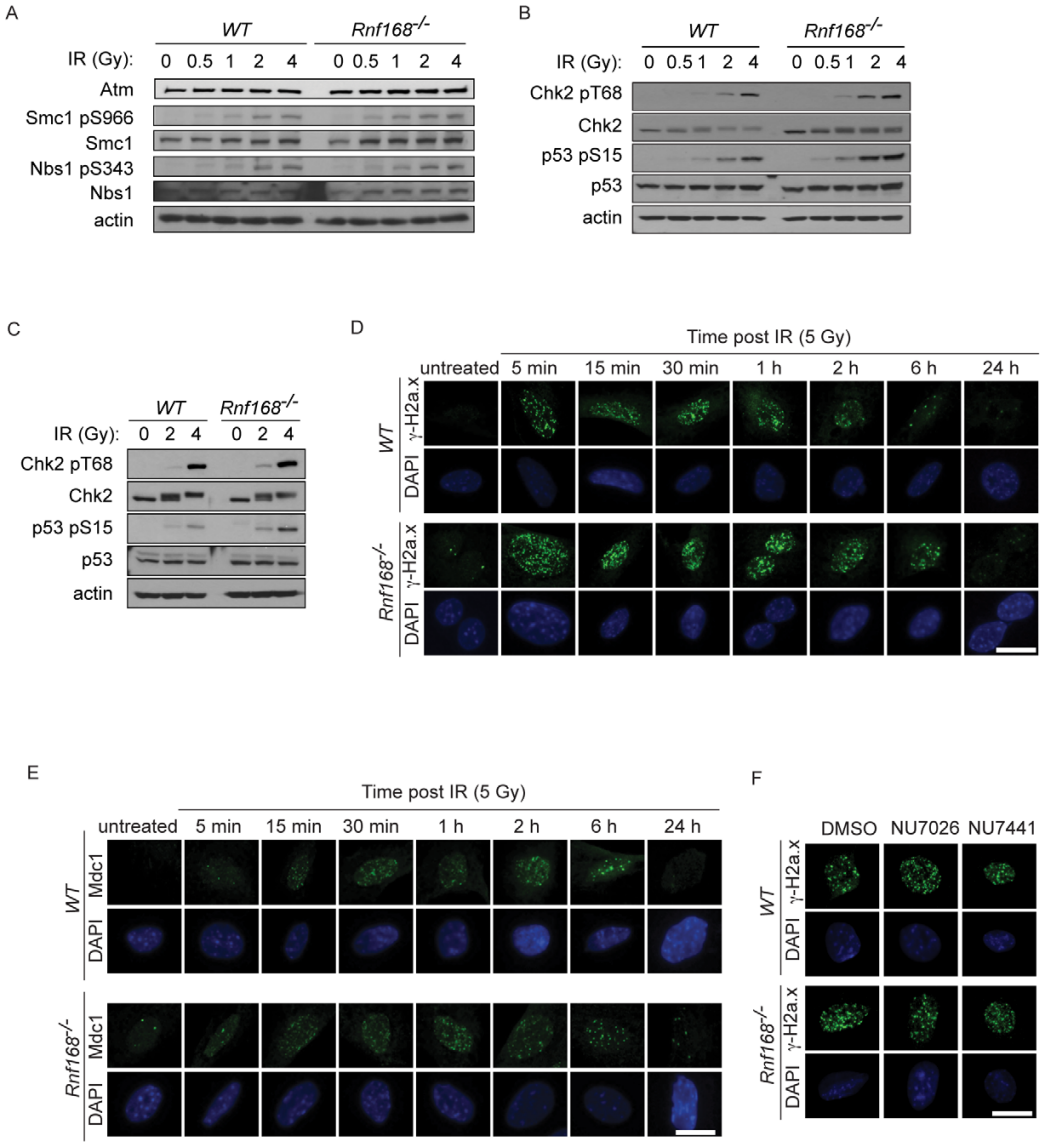
Atm signaling pathway is not affected in the absence of Rnf168. (A–C) IB analysis of untreated or irradiated *WT* and *Rnf168^−/−^* thymocytes (A and B) or MEFs (C). Cells were irradiated as indicated and examined 1 hour post-IR by immunoblotting using the indicated antibodies. Representative data are shown from three independent experiments. (D) Representative micrographs of MEFs stained with anti γ-H2a.x antibody and DAPI. *Rnf168^−/−^* and *WT* MEFs were untreated or exposed to 5 Gy of IR and fixed at the indicated time after IR. Bars, 20 µm. Three independent experiments were performed. (E) Representative micrographs of MEFs stained with anti-Mdc1 antibody and DAPI. *Rnf168^−/−^* and *WT* MEFs were untreated or exposed to 5 Gy of IR and fixed at the indicated times post IR. Three independent experiments were performed. Bars, 20 µm. (F) Representative micrographs of MEFs treated with DNA-PK inhibitors and stained with anti γ-H2a.x antibody and DAPI. *Rnf168^−/−^* and *WT* MEFs were treated with DNA-PK inhibitors (NU7026 or NU7441) and cultured for 1 hour. MEFs were left untreated or exposed to 5 Gy of IR and fixed 2 hours post-IR. Three independent experiments were performed. Bars, 20 µm.

To examine whether Rnf168 deficiency affects the activation of Atm pathway, *WT* and *Rnf168^−/−^* thymocytes were irradiated *ex vivo*. One hour post-IR, the protein levels of total Atm, as well as the expression and phosphorylation levels of its downstream targets, were assessed by immunoblot (IB) analysis. The protein levels of Atm were similar between *Rnf168^−/−^* and *WT* thymocytes in response to IR (Figure 3A). Phosphorylation levels of the Atm substrates Smc1 (serine 966), Nbs1 (serine 343), Chk2 (threonine 68) and p53 (serine 15), were similar or slightly higher in *Rnf168^−/−^* thymocytes compared to *WT* controls (Figure 3A and 3B). IR also induced a slightly higher phosphorylation level of Chk2 (threonine 68) and p53 (serine 15) in *Rnf168^−/−^* MEFs compared to *WT* controls (Figure 3C). We also observed increased γ-H2a.x and Mdc1 foci formation in untreated and irradiated *Rnf168^−/−^* MEFs compared to *WT* controls (Figure 3D and 3E, Figure S3C and S3D). In addition, γ-H2a.x- and Mdc1- IRIF remained visible longer in irradiated *Rnf168^−/−^* MEFs compared to *WT* controls, suggesting defective DSB repair in the absence of Rnf168. As both Atm and DNA-PK can phosphorylate H2a.x at serine 139 in response to IR [31], we examined the effects of inhibition of DNA-PK on γ-H2a.x IRIF in *WT* and *Rnf168^−/−^* MEFs. The levels of γ-H2a.x IRIF in irradiated *WT* and *Rnf168^−/−^* MEFs were not affected by DNA-PK inhibitors (Figure 3F). Collectively, these data indicate that Rnf168 is dispensable for the activation of ATM-Chk2-p53 pathway and that Atm signaling pathway is unaffected in *Rnf168^−/−^* cells.

### Age-dependent requirement for Rnf168 in spermatogenesis

**Figure 4:**
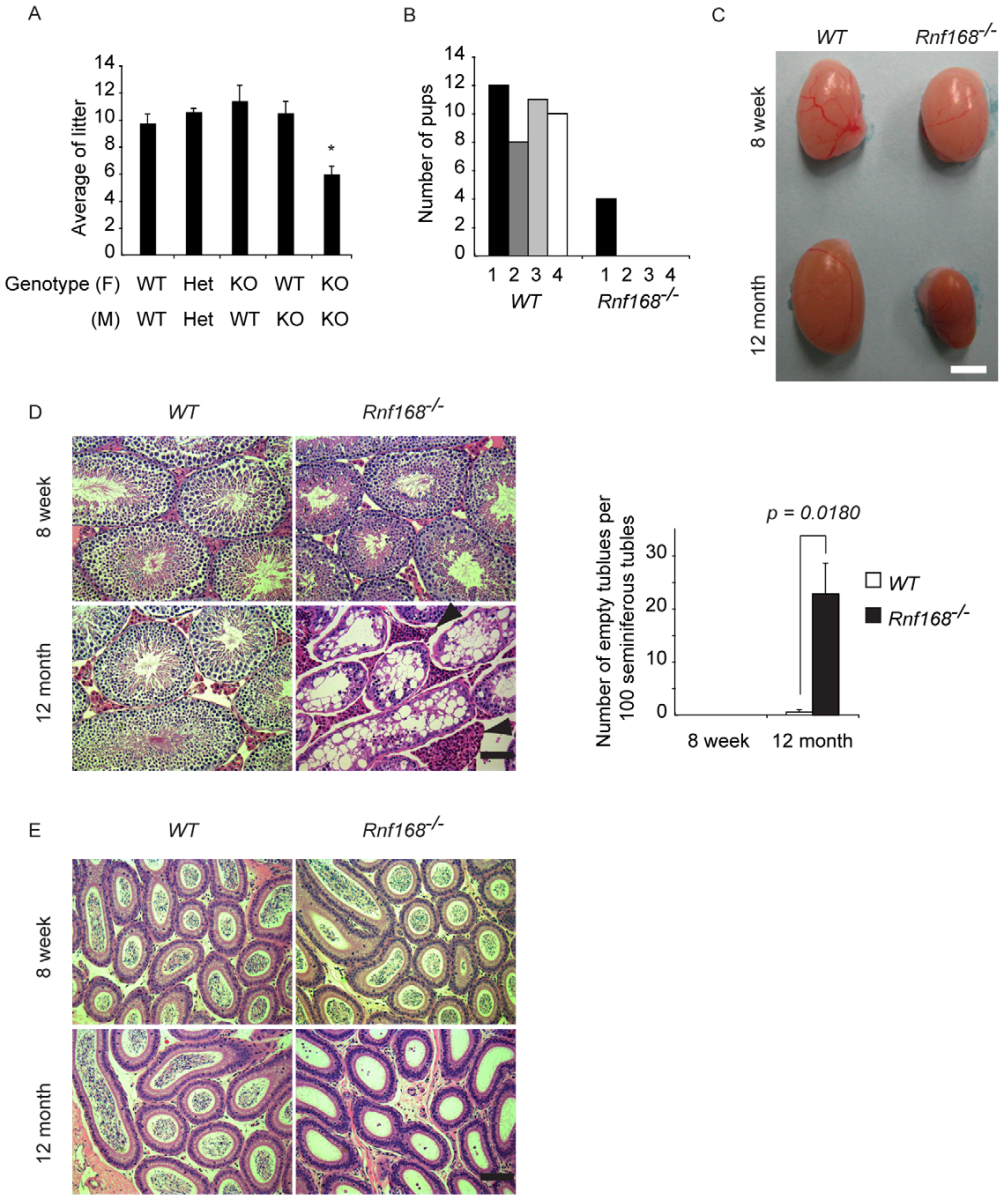
Impaired long-term spermatogenesis in *Rnf168^−/−^* mice. (A) Average litter sizes from intercrosses of 8-week-old mice with the indicated genotypes are shown [WT (F)×WT (M); n = 4, Het (F)×Het (M); n = 5, KO (F)×WT (M); n = 5, WT (F)×KO (M); n = 4, KO (F)×KO (M); n = 3. F = female, M = male, Het = Heterozygote (*Rnf168^+/−^*), KO = knockout (*Rnf168^−/−^*)]. (B) Twelve-month-old *WT* or *Rnf168^−/−^* males were mated with 8-week-old females. Number of pups from breeding 4 pairs of each genotype is shown. (C) Comparison of testes size in 8-week-old and 12-month-old *WT* or *Rnf168^−/−^* males. Bar, 3 mm. (D) Sections of testes stained with hematoxylin-eosin (left panels). Arrowheads indicate Leydig cells. Bar, 100 µm. The numbers of empty tubules per 100 seminiferous tubules are graphed (right panel). Data are presented as the mean ± SEM (*WT* n = 3; *Rnf168^−/−^* n = 3). *p<0.05. (E) Sections of epididymis stained with hematoxylin-eosin. Bar, 100 µm. Three mice of each genotype were analyzed.

H2a.x, Mdc1 and Rnf8 are required for DDR upstream to Rnf168, and are essential for male fertility [10], [27], [30], [32]–[35], whereas *53bp1* homozygous mutant males are fertile [28]. We therefore investigated the fertility of *Rnf168^−/−^* mice. *Rnf168^−/−^* males and females were fertile at 8 weeks of age, albeit their intercrosses produced smaller litters (6.0 pups±0.6) compared to *WT* littermates (9.8 pups±0.7, *p<0.05*) (Figure 4A). In contrast, 12-month-old *Rnf168^−/−^* males showed either a significantly reduced fertility compared to *WT* littermates or were completely infertile (Figure 4B). Testicular sizes were comparable between 8-week-old *Rnf168^−/−^* and *WT* littermates, whereas at 12 months of age, *Rnf168^−/−^* males showed reduced testicular size compared to *WT* littermates (Figure 4C). Histological examination of testes from 8-week-old *Rnf168^−/−^* males indicated no abnormalities compared to *WT* littermates (Figure 4D). However, testes from aged *Rnf168^−/−^* mice displayed signs of testicular degeneration and atrophy as evidenced by the reduced number or lack of spermatids in seminiferous tubules, the increased vacuolization of germ cells and the prominent Leydig cells compared to *WT* littermates (Figure 4D). The amount of sperm was also drastically decreased in the epididymis of 12-month-old *Rnf168^−/−^* males compared to controls (Figure 4E). All together, these data indicate that Rnf168 is required for spermatogenesis in an age-dependent manner.

### Inactivation of Rnf168 impairs immunoglobulin class switch recombination and results in immunodeficiency

The proportion of B and T lymphocytes in one RIDDLE syndrome patient was reported to be within the normal range; however, this patient was immunodeficient and exhibited low levels of serum immunoglobulin G (IgG) [24]. To investigate the effect of Rnf168 deficiency on lymphocyte maturation, we first examined bone marrow (BM) from 6–8-week-old *Rnf168^−/−^* mice and their *WT* littermates. BM cellularity and the number of Pro- and Pre-B-cells were not affected by Rnf168 deletion (Figure S4A). Similarly, examination of the number of splenocytes, LN cells and their subpopulations also showed no significant difference between *Rnf168^−/−^* mice and *WT* controls (Figure S4B and S4C).

**Figure 5:**
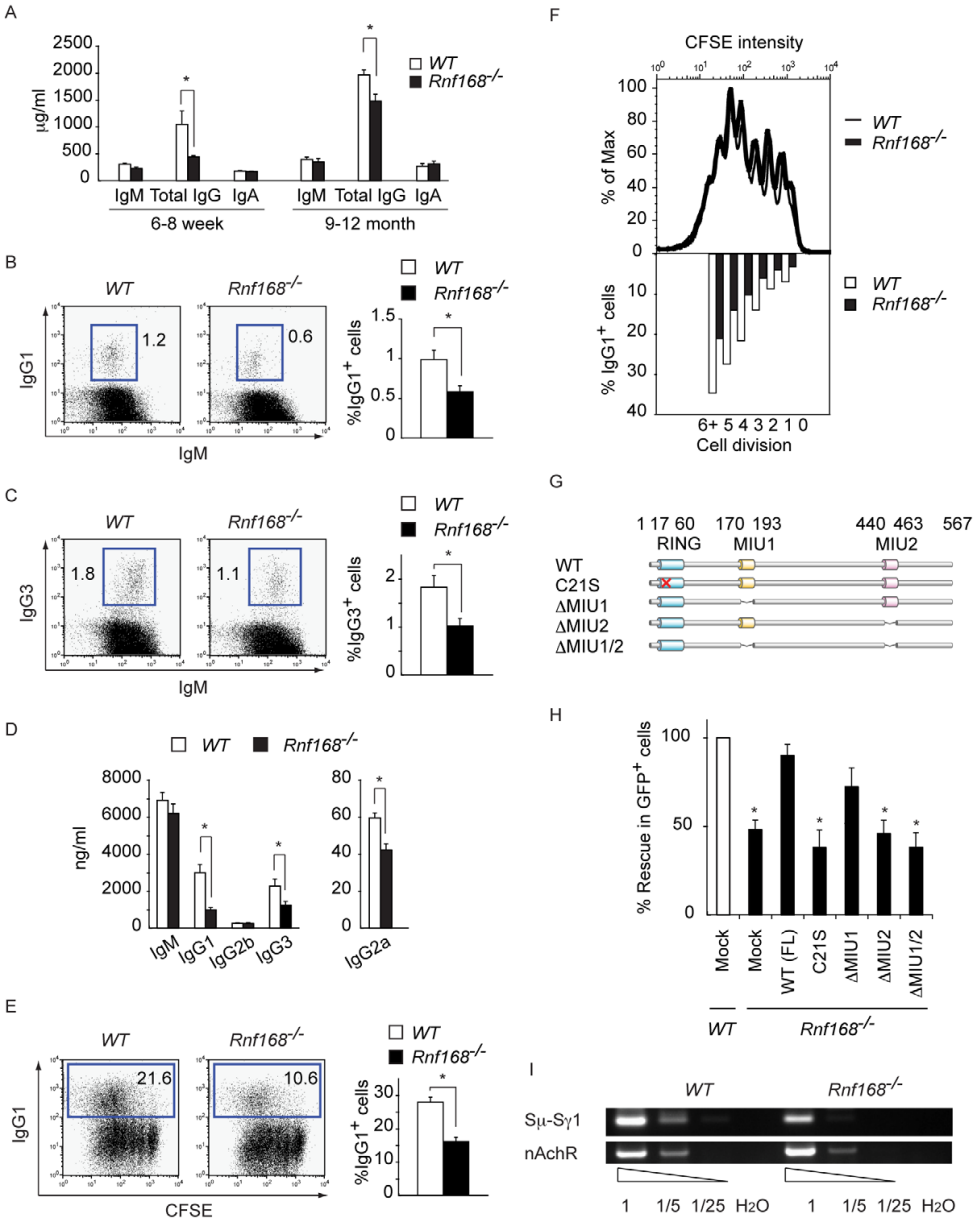
Impaired class switch recombination in *Rnf168^−/−^* mice. (A) Serum immunoglobulin levels in 6–8-week-old and 9–12-month-old *Rnf168^−/−^* mice. The data are presented as the mean ± SEM (n = 3–5). (B and C) FACS analysis of IgG1 (B) or IgG3 (C) and IgM expression on B220^+^ B-cells (left panels) and average percentages of IgG1^+^ or IgG3^+^ B-cells (right panels) from Peyer's patches. Three independent experiments were performed. (D) Secreted immunoglobulins were analyzed in the supernatants of B-cell cultures after 4 days of *in vitro* stimulation with LPS with or without IL-4. The data are presented as the mean ± SEM from three independent experiments. (E) FACS analysis of IgG1 expression on CFSE labeled B-cells stimulated with anti-CD40 plus IL-4 for 4 days (left panels) and average percentages of IgG1 switched cells (right panel). Five independent experiments were performed. (F) CFSE staining profiles of B-cells stimulated with anti-CD40 antibody plus IL-4 for 4 days (upper panel) and percentages of IgG1^+^ cells that had undergone the indicated number of cell divisions (lower panel). Representative data are shown from three independent experiments. (G) Schematic representations of *WT* and mutants *Rnf168* cloned into the MSCV-IRES-GFP vector. (H) B-cells infected with the indicated ecotropic retroviruses [MSCV-mutated or full-length (FL) Rnf168-IRES-GFP] were stimulated with LPS plus IL-4 for 4 days, and the level of CSR to IgG1 was examined by FACS. Data are presented as the mean ± SEM of four independent experiments. * indicates *p<0.05* compared to *WT*. (I) Representative DC-PCR for Sμ-Sγ1 recombination is shown from three independent experiments. *nAchR* served to normalize for the amount of input DNA. Fivefold serial dilutions were used as templates. H_2_O: no input DNA.

The repair of programmed DSBs is essential for class switch recombination (CSR) of immunoglobulins [36], [37]. Failure to initiate, signal, or repair these programmed DSBs will lead to defective CSR and immunodeficiency. To assess the role of Rnf168 in *Ig heavy chain* (*Igh*) class switching *in vivo*, we evaluated the concentrations of serum Ig isotypes in *Rnf168^−/−^* mice and *WT* littermates. Total serum IgG concentrations were significantly lower in 6–8-week-old *Rnf168^−/−^* mice and in 9–12-month-old *Rnf168^−/−^* mice compared to age-matched *WT* littermates (Figure 5A). To further examine the *in vivo* role of Rnf168 in CSR, we determined the portion of IgG1- and IgG3-expressing B-cells in Peyer's patches from *Rnf168^−/−^* mice and *WT* littermates. B-cells in the Peyer's patches constantly encounter the gut flora, and, therefore, they exhibit elevated levels of CSR [38]. Peyer's patches from *Rnf168^−/−^* mice showed decreased representation of IgG1- and IgG3-expressing B-cells compared to *WT* littermates (*p<0.05*; Figure 5B and 5C).

We further examined the role Rnf168 plays in CSR for various Igh isotypes using purified splenic B-cells from *Rnf168^−/−^* mice and *WT* littermates. When B-cells were activated in culture with LPS±IL4, Rnf168-deficient B-cells displayed a significantly reduced secretion of IgG1, IgG2a and IgG3 compared to *WT* controls (Figure 5D). We also stimulated *in vitro* purified splenic B-cells with anti-CD40 or LPS in combination with IL-4, and examined the levels of CSR to IgG1. To analyze the efficiency of CSR in each cell division, we used FACS analysis to assess the expression of surface IgG1 on CFSE-labeled stimulated B-cells (Figure 5E and 5F, Figure S4D and S4E). The portion of B-cells expressing surface IgG1 post activation was significantly reduced in *Rnf168^−/−^* mice (13.2±1.1%) compared to *WT* controls (28.3±0.8%, *p<0.05*) (Figure 5E). Examination of the CFSE dilution profiles indicated similar proliferation levels of mature B-cells from *WT* and *Rnf168^−/−^* mice (Figure 5F and Figure S4E). In addition, the efficiency of CSR in each cell division was decreased in activated *Rnf168^−/−^* B-cells compared to *WT* B-cells (Figure 5F). These data indicate that CSR defects in *Rnf168^−/−^* B-cells were directly due to the loss of Rnf168 function, rather than a result of proliferation defects of the mutant B-cells.

The RING finger domain of RNF168 is critical for its E3 ligase activity, while its MIU2 domain is important for its recruitment to DSB sites [20], [22]. To further explore the role of Rnf168 in CSR, we generated deletion or point mutant *Rnf168* constructs using the pMSCV retroviral vector. Purified B-cells from *WT* or *Rnf168^−/−^* mice were activated with LPS and IL-4 and then retrovirally transduced with the various Rnf168-expression constructs. These cells were then examined for functional rescue of the impaired CSR by WT or mutants Rnf168 (Figure 5G, 5H and Figure S4F). *Rnf168^−/−^* B-cells, infected with pMSCV expressing either *WT Rnf168* or *Rnf168* with its MIU1 domain deleted (ΔMIU1), rescued IgG1 CSR to the level of *WT* B-cells. However, *Rnf168^−/−^* B-cells infected with retroviruses expressing *Rnf168* with mutated RING finger domain (C21S; cysteine 21 residue to serine), deleted MIU2 domain (ΔMIU2) or deleted MIU1 and MIU2 domains (ΔMIU1/2), showed no rescue of their CSR defects.

We next examined the switch recombination directly by using a quantitative digestion-circularization PCR (DC-PCR) assay [39]. These analyses demonstrated lower frequency of Sμ-Sγ1 switch recombination in activated *Rnf168^−/−^* B-cells compared to *WT* B-cells (Figure 5I and Figure S4G).

**Figure 6:**
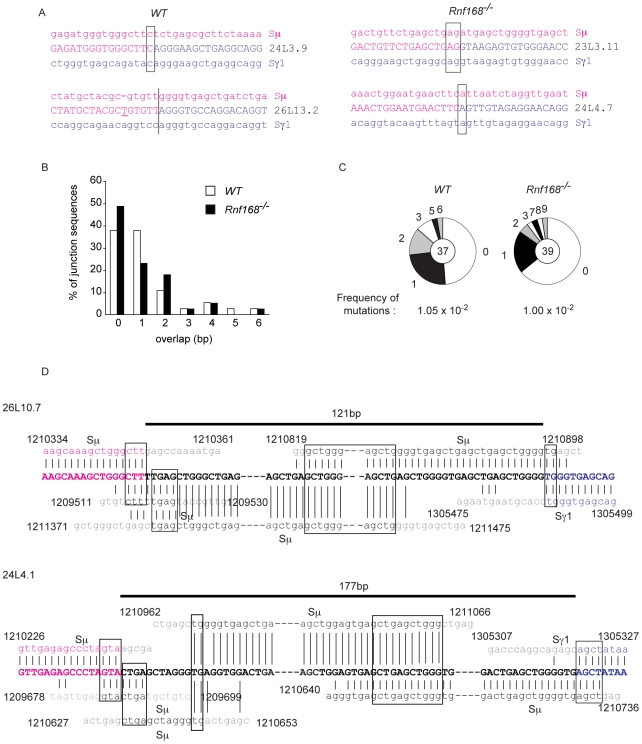
Analysis of the effect of Rnf168 inactivation on Sμ-Sγ1CSR junctions. (A) Analysis of Sμ-Sγ1 CSR junctions. Overlap was determined by identifying the longest region at the switch junction of perfect uninterrupted donor/acceptor identity. No significant differences were observed. Representative data are shown from more than thirty clones in three independent experiments. (B) Purified splenic B cells were stimulated with LPS and IL-4 for 4 days. Genomic DNA was amplified by PCR and Sμ-Sγ1 junctions were sequenced. The percentage of junctions with the indicated nucleotide overlap is indicated (37 sequences from three *WT* mice and 39 sequences from three *Rnf168^−/−^* mice were analyzed). (C) Mutations in B-cells stimulated with LPS plus IL-4 for 4 days. Mutations near the Sμ-Sγ1 junctions (±50 bp) and frequencies of mutations are shown (37 sequences from three *WT* mice and 39 sequences from three *Rnf168^−/−^* mice were analyzed). The numbers of observed mutations are indicated in the periphery of the circular charts. (D) Sμ/Sγ1 junctions with unusual insertions obtained from *Rnf168^−/−^* B-cells. Sμ/Sγ1 sequences are shown in bold. The Sμ and Sγ1 [NT_114985.2 (strain 129/SvJ)] germline sequences are shown above or below each junction sequence. Lower-case letters indicate insertions. (|) indicates identity between nucleotides. Homology at the junctions is boxed. Two clones, 26L10.7 and 24L4.1, were obtained from independent experiments. 37 sequences from three *WT* mice and 39 sequences from three *Rnf168^−/−^* mice were analyzed.

The DNA sequences of CSR junctions can offer additional insights into the mechanistic defects of the joining process [40]. For instance, B-cells with homozygous mutations in *Xrcc4* or *DNA Ligase IV* undergo impaired CSR and form CSR junctions with increased sequence microhomology, indicating the use of an alternative joining pathway in these mutant cells [41], [42]. Previous studies reported that B-cells deficient for H2a.x or 53bp1 show no significant differences in the extent of microhomology at the switch junctions [40], [43]–[45]. To examine the effects of Rnf168 deficiency on the nucleotide composition of the switch junction regions, we cloned and sequenced the Sμ-Sγ1 junctions from *Rnf168^−/−^* and *WT* B-cells stimulated *in vitro* with LPS and IL-4. Such analysis revealed no significant differences in the extent of donor/acceptor homology (Figure 6A and 6B), in the frequency of mutations (Figure 6C) nor in the average length of overlaps (Table S2). Interestingly, in contrast to *WT* controls, 5.1% of the examined CSR junctions in *Rnf168^−/−^* B-cells were found to harbor long insertions (Figure 6D). Similar long insertions of nucleotides were observed in 2 out of 40 (5%) CSR junctions of *53bp1^−/−^* B-cells but not in *Atm^−/−^*, *H2a.x^−/−^*, *Nbs1^−/−^* or *WT* B-cells [40], [43], [44], [46]. These data therefore suggest that Rnf168 contributes to the 53bp1 function in the synapsis of DNA ends.

Collectively, these data demonstrate that Rnf168 is required for *in vitro* and *in vivo* CSR, and its inactivation in mutant mice leads to immunodeficiency, which parallels the symptoms of the RIDDLE syndrome. Furthermore, we demonstrate that the RING finger and MIU2 domains of Rnf168 are indispensable for efficient CSR. Finally, our data also indicate that, similar to *53bp1^−/−^* B-cells, *Rnf168^−/−^* B-cells display increased frequency of long nucleotide insertions at the CSR junctions.

### A role for Rnf168 in long-range V(D)J recombination

*53bp1^−/−^* mice exhibit impaired early thymocyte development, an accumulation of early CD4^−^CD8^−^ double negative (DN) thymocytes at the DNIII stage (CD44^−^CD25^+^), reduced TCRβ expression, and lower frequency of TCRγδ^+^ cells compared to *WT* littermates [28], [47], [48]. In contrast to the pronounced decrease of TCRβ expression in *53bp1^−/−^* thymocytes, expression of TCRβ was not significantly affected in thymocytes deficient for *H2a.x*, *Mdc1* or *Rnf8* [30], [34], [47].

**Figure 7:**
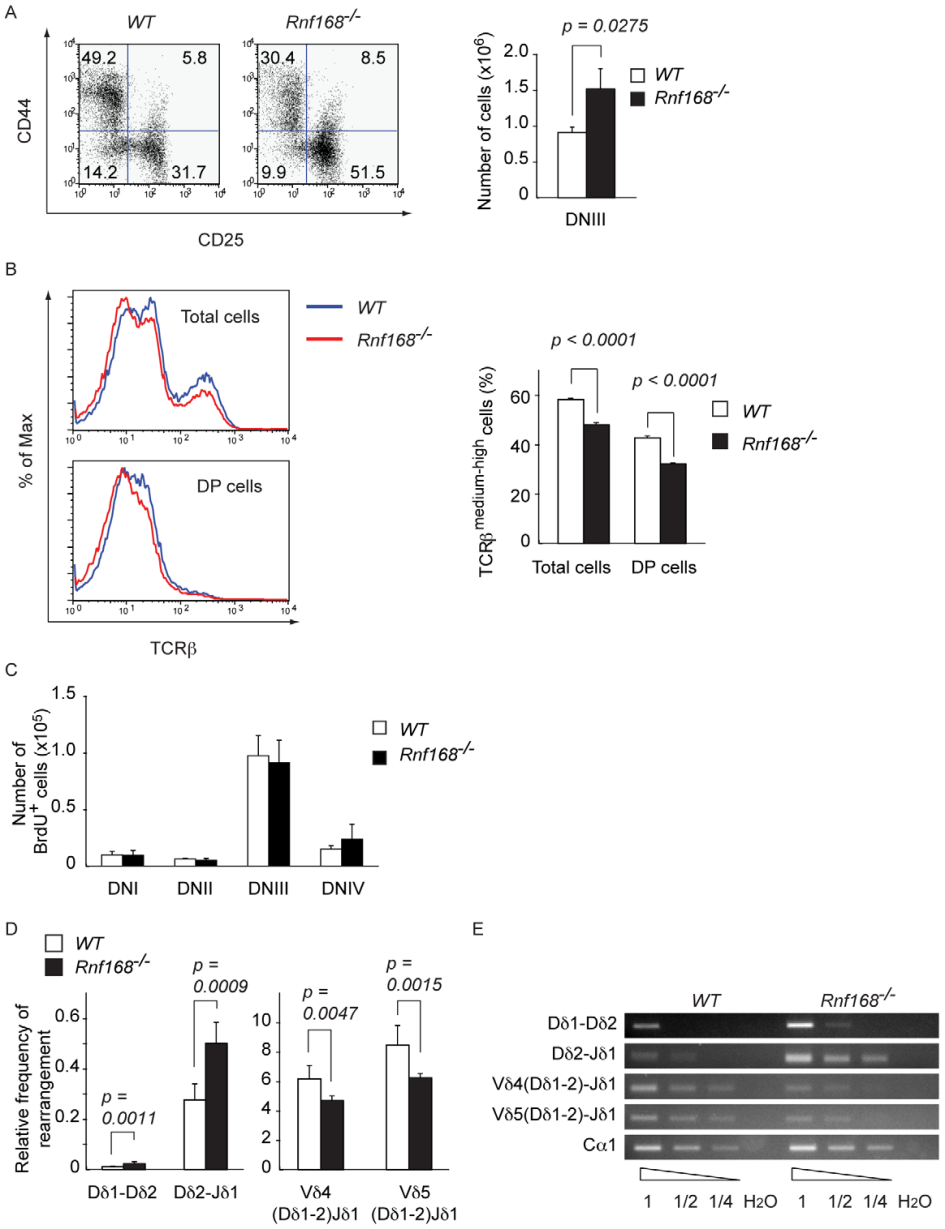
Effect of Rnf168 deficiency on thymocyte development, TCRβ expression, and long-range V(D)J recombination. (A) Flow cytometric analyses of DN thymocytes from *WT* and *Rnf168^−/−^* mice and average number of CD4^−^CD8^−^CD44^−^CD25^+^ (DNIII) cells. Data are presented as the mean ± SEM (n = 13 for each genotype). *p<0.05. (B) Expression levels of TCRβ in total thymocytes and CD4^+^CD8^+^ (DP) cells. (C) Histograms of the mean number of BrdU^+^ cells in each subpopulation of DN thymocytes from *WT* (n = 3) or *Rnf168^−/−^* (n = 3) mice. Data are presented as the mean ± SEM. (D) Relative frequency of *Tcr*δ locus rearrangements in total thymocytes. Quantitative assessment of genomic DNA rearrangements of Dδ1 to Dδ2, Dδ2 to Jδ1, and Vδ4 and Vδ5 to (D)Jδ1 genes were performed by real-time quantitative PCR and normalized to the signal of the non-rearranging DNA 3′ of Jδ2. Data are presented as the mean ± SEM (*WT* n = 9; *Rnf168^−/−^* n = 7). **p<0.005. (E) Representative primary PCR data for genomic DNA rearrangements of Dδ1 to Dδ2, Dδ2 to Jδ1, and Vδ4 and Vδ5 to (D)Jδ1.

FACS analysis of thymocytes from *Rnf168^−/−^* mice showed increased frequency of DN thymocytes compared to *WT* littermates (Figure S5A). Examination of DN subpopulations indicated a significantly impaired transition of *Rnf168^−/−^* early thymocytes at the DNIII stage (Figure 7A, left panel). In addition, total number of DNIII thymocytes in *Rnf168^−/−^* mice (1.5×10^6^±2.8×10^5^) was significantly increased compared to *WT* littermates (1.1×10^6^±1.9×10^5^, *p = 0.025*) (Figure 7A, right panel). *Rnf168^−/−^* thymocytes also displayed a mild decrease in their expression of TCRβ compared to *WT* controls (Figure 7B) and the frequency of TCRγδ^+^ thymocytes in *Rnf168^−/−^* mice was significantly reduced compared to *WT* controls (Figure S5B; *WT* 0.24±0.21%, *Rnf168^−/−^* 0.20±0.01%, *p<0.005*). Productive rearrangement of the TCRβ locus takes place during the DNIII to DNIV transition and leads to pre-TCR expression [49]–[51]. Only cells expressing functional pre-TCR undergo exponential expansion, a process referred to as β-selection [49]–[51]. To assess the proliferation level of each DN thymocyte subpopulations, we examined *in vivo* thymocyte BrdU uptake in *WT* and *Rnf168^−/−^* mice. The number of BrdU^+^ cells was not affected in *Rnf168^−/−^* mice compared to *WT* littermates (Figure 7C). Therefore, the increased number of DNIII thymocytes, the decreased expression of TCRβ and the reduced number of TCRγδ cells in *Rnf168^−/−^* thymocytes were not due to proliferative defects. However, it remains possible that developmental defects can also contribute to these differences.

Loss of function of 53bp1 results in impaired *Tcr* locus integrity due to dysfunctional long-range V(D)J rearrangement [47]. To examine whether Rnf168 facilitates joining of distal gene segments during V(D)J recombination, we performed quantitative PCR assays of the partial (Dδ2-Jδ1 and Dδ1-Dδ2) and complete (Vδ5-Dδ1 and Vδ4-Dδ1) rearrangements. We observed that short-range rearrangements were more abundant in *Rnf168* ^−/−^ thymocytes compared to *WT* controls (Figure 7D, left panel, Figure 7E, and Figure S5C). On the other hand, complete Vδ-to-DδJδ recombination was significantly reduced in *Rnf168* ^−/−^ thymocytes compared to *WT* controls (Figure 7D, right panel, Figure 7E and Figure S5C).

These data support a role for Rnf168 in early thymocyte development and indicate that long-range V(D)J recombination is impaired in the absence of Rnf168.

### Role of Rnf168 in maintaining genomic integrity and suppressing cancer

**Figure 8:**
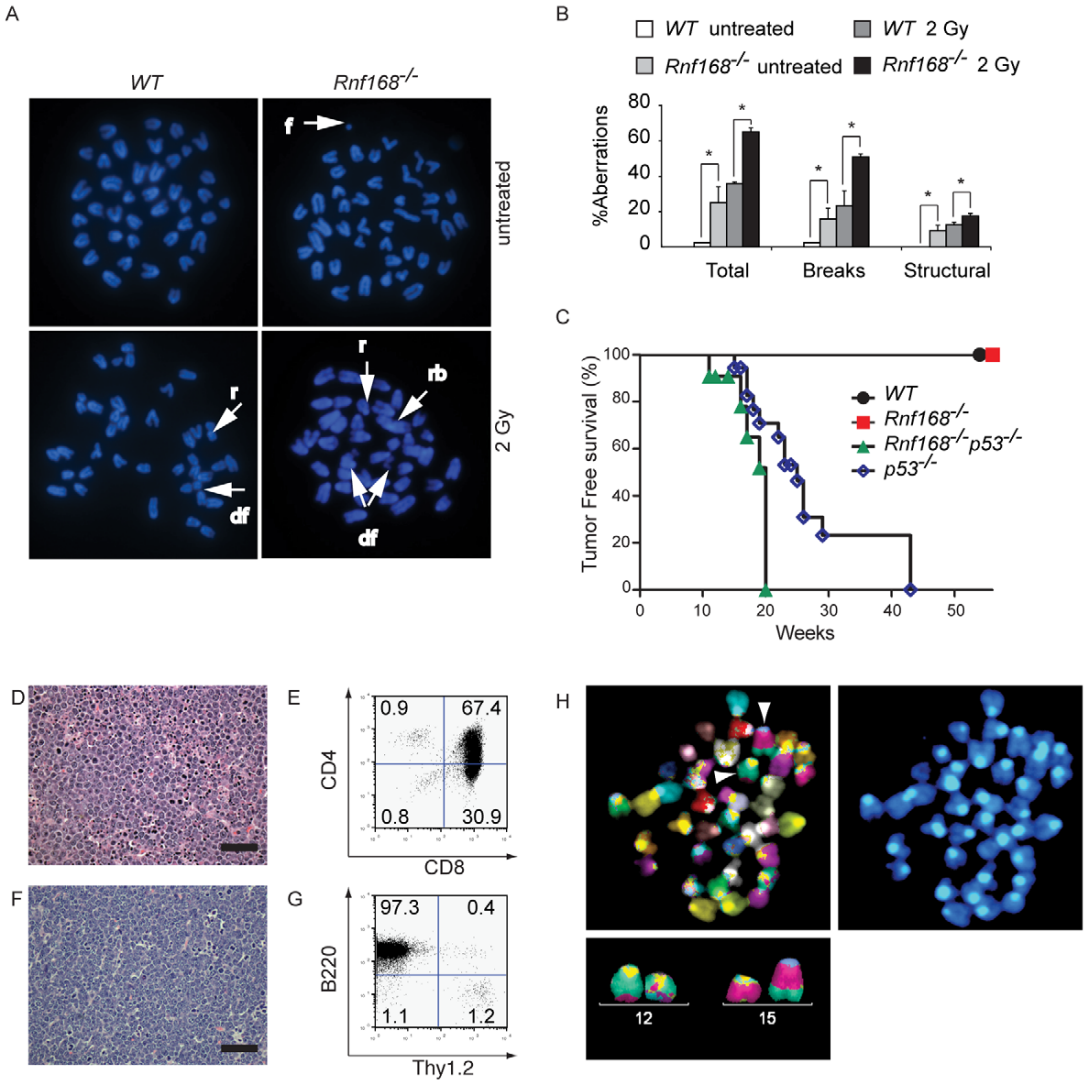
Rnf168 maintains genomic integrity and suppresses cancer. (A and B) Metaphase spread analysis of *Rnf168^−/−^* and *WT* B-cells. Representative data (A) and the percentage of aberrations (B) are shown. Three independent experiments were performed. A minimum of 40 metaphase spreads of untreated or irradiated *Rnf168^−/−^* and *WT* cells were analyzed. *p<0.05. f = acentric fragment, r = ring, rb = Robertsonian translocation, df = double acentric fragment. (C) Kaplan Meier tumor-free survival analysis for cohorts of *WT* (n = 56), *Rnf168^−/−^* (n = 50), *p53^−/−^* (n = 18) and *Rnf168^−/−^p53^−/−^* (n = 11) mice. A statistically significant difference was observed between the tumor-free survival of *Rnf168^−/−^p53^−/−^* and *p53^−/−^* mice (*p* = 0.0096, log-rank test). (D and E) H&E staining (D) and FACS analysis (E) of a thymoma from an *Rnf168^−/−^p53^−/−^* mouse. (F and G) H&E staining (F) and FACS analysis (G) of a B-cell lymphoma (B220^+^) from an *Rnf168^−/−^p53^−/−^* mouse. (H) Chromosomal translocations observed in an *Rnf168^−/−^p53^−/−^* lymphoma. Clonal reciprocal chromosomal translocations t(12;15) and t(15;12) are shown. Scale Bars: 50 µm; (D and F).

Defective signaling or repair of DSBs impairs genomic integrity [4], [52]. For instance, inactivation of Atm, H2a.x, Mdc1, Rnf8, 53bp1 or Brca1 results in increased genomic instability [10], [27], [28], [30], [48], [53]–[56]. To evaluate the effect of Rnf168 inactivation on genomic integrity, we examined for chromosomal aberrations in metaphase spreads of LPS activated *Rnf168^−/−^* and *WT* B-cells (Figure 8A and 8B). The level of spontaneous chromosomal aberrations was elevated in *Rnf168^−/−^* cells (25±9.0%) compared to *WT* cells (2.5±0%, *p<0.05*). Interestingly, *Rnf168^−/−^* cells demonstrated increased frequencies of DNA breaks (15.8±6.0%) and structural chromosomal aberrations (9.2±3.0%) compared to *WT* controls (DNA breaks: 2.5±0%, *p<0.05*, structural chromosomal aberrations: 0%, *p<0.05*). Increased spontaneous genomic instability in *Rnf168^−/−^* cells was consistent with the elevated frequency of spontaneous γ-H2a.x- and Mdc1-IRIF in *Rnf168^−/−^* cells (Figure 3D and 3E). In response to IR (2 Gy), the level of genomic instability was further elevated in *Rnf168^−/−^* cells (65±2.2%) compared to *WT* controls (35.8±1%, *p<0.05*). The frequencies of IR-induced breaks (50.8±1.7%) and structural chromosomal aberrations (17.5±1.4%) were greater in *Rnf168^−/−^* cells compared to *WT* cells (DNA breaks: 23.3±8.4%, *p<0.05*; structural chromosomal aberrations: 12.5±1.4%, *p<0.05*). Therefore, Rnf168 deficiency leads to increased spontaneous and IR-induced genomic instability.

Mice deficient for Mdc1, Rnf8, 53bp1 or Brca1 have increased cancer susceptibility likely due to their elevated levels of genomic instability [28], [30], [54]–[57]. To examine whether inactivation of Rnf168 predisposes for tumor development, we monitored cohorts of *Rnf168^+/+^* (n = 56) and *Rnf168^−/−^* (n = 52) mice for a period of 12 months; however, none of the monitored *Rnf168^−/−^* mice developed tumors during the 1 year period (Figure 8C).

Activated *Rnf168^−/−^* B cells displayed increased genomic instability, but the activation of Atm-Chk2-p53 pathway remained intact in the absence of Rnf168. These findings prompted us to examine whether this pathway has any role in preventing tumorigenesis in *Rnf168^−/−^* mice. To do so, we generated mice lacking both Rnf168 and p53. *Rnf168^−/−^p53^−/−^* mice showed no gross overall developmental defects compared to their single mutant littermates. Cohorts of *Rnf168^−/−^p53^−/−^* mice (n = 11) and *p53^−/−^* mice (n = 18) were monitored for survival. Interestingly, we observed a significant decrease in the tumor free survival of *Rnf168^−/−^p53^−/−^* mice compared to *p53^−/−^* controls (*p* = 0.0096, log-rank test).

Consistent with previous studies [58]–[60], the majority of tumors developed by *p53^−/−^* mice were thymomas (Table S3). However, a different spectrum of tumors was observed in *Rnf168^−/−^p53^−/−^* mice, including thymomas (Figure 8D and 8E), B-cell lymphomas (Figure 8F and 8G), hemangiosarcoma (Figure S6A and S6B) and sarcoma (Figure S6C and S6D). *Rnf168^−/−^p53^−/−^* thymomas and B-cell lymphomas were found to infiltrate various non-lymphoid organs including lung, liver and salivary glands (Figure S6E–S6H).

To examine chromosomal translocations in these tumors, we performed multicolor fluorescence *in situ* hybridization (mFISH) experiments using three B-cell lymphomas and one thymoma from *Rnf168^−/−^p53^−/−^* mice. Whereas *p53^−/−^* thymic lymphomas have been reported to rarely harbor chromosomal translocations [61], [62], two of the three examined *Rnf168^−/−^p53^−/−^* B-cell lymphomas carried clonal reciprocal translocations between chromosomes 12 and 15 [t(12;15) and t(15;12)] (Table S4 and Figure 8H). A clonal non-reciprocal translocation, t(9;11), was also observed in one of the examined *Rnf168^−/−^p53^−/−^* B-cell lymphomas (Table S4). Finally, complex chromosomal abnormalities were observed in the examined *Rnf168^−/−^p53^−/−^* thymoma (Table S4).

Collectively, these data demonstrate that Rnf168 is important for maintaining genomic integrity, and it cooperates with p53 in suppressing tumorigenesis.

## Discussion

The *RNF168* gene is located on human chromosome 3q29, and encodes a novel E3 ligase that plays an important role in the signaling of DSBs [20]–[22]. Interestingly, mutations of *RNF168* have recently been identified as the genetic defects leading to RIDDLE syndrome in humans [22], [24]. In order to investigate the *in vivo* functions of this E3 ligase, we have generated a mouse model for *Rnf168* mutation.

Similar to mice mutant for its upstream DSBs signaling proteins, including H2a.x, Mdc1 and Rnf8, *Rnf168^−/−^* mice are viable [10], [27], [30], [34], [35]. In contrast to *Rnf8^−/−^* mice that display reduced number of lymphocytes [30], [34], no defect in T- or B-cell numbers was observed in *Rnf168^−/−^* mice. The normal number of lymphocytes in *Rnf168^−/−^* mice is in accordance with the lack of lymphopenia in the patient with RIDDLE syndrome [24].

Initial recruitment of 53bp1 to DSB sites is not affected in the absence of H2a.x, Mdc1 or Rnf8 [10], [29], [30], [63]–[65]. However, inactivation of Rnf168 in MEFs completely abolishes both the transient and retained 53bp1 IRIFs. These data highlight the importance of Rnf168 for the initial recruitment of 53bp1 to DSB sites, and indicate that this function of Rnf168 is independent of H2a.x, Mdc1 and Rnf8.

Immunodeficiency, a hallmark for RIDDLE syndrome, is characterized by normal ratio of T and B cells, but low levels of serum IgG likely due to impaired CSR [24], [66]. Interestingly, defective CSR was also observed in B-cells deficient for Atm, H2a.x, Mdc1, Rnf8 or 53bp1 [10], [27], [30], [34], [39], [44], [45], [66], [67].

*Rnf168^−/−^* mice showed reduced serum immunoglobulin levels, although this defect was milder compared to *Rnf8^−/−^* mice [30]. The impaired B-cell development observed in *Rnf8^−/−^* mice [30], [34], but not *Rnf168^−/−^* mice, might contribute to the more pronounced reduction in non-IgM classes in *Rnf8^−/−^* mice compared to *Rnf168^−/−^* mice. Although *Rnf168^−/−^* mice showed a normal IgA levels in serum, they demonstrated impaired CSR for various IgG isotypes, thus reproducing the CSR defect associated with RIDDLE syndrome [24]. Defective CSR in *Rnf168^−/−^* mice is also consistent with impaired CSR following the knockdown of Rnf168 in the B-cell line CH12F3-2 [66].

Recent studies have demonstrated the functions of 53BP1 in DNA damage signaling and repair [40], [45], [47], [67]–[70]. 53BP1 promotes and maintains synapsis during V(D)J recombination [40], [47]. Prior to recombination, 53BP1 constitutively associates with chromatin, supports long-range tethering of recombination signal sequence synapsis, and prevents extensive DNA end resection [47], [69], [70]. 53BP1 is also important for early T-cell development and for CSR [47]. In this study, we show that inactivation of Rnf168 impairs long-range V(D)J recombination in both early T-cell development and in B-cell class switch junction, suggesting that Rnf168 is required for proper synapsis of distal DSBs.

Impaired signaling of DSBs often results in male infertility as observed in *H2a.x^−/−^* [32], *Mdc1^−/−^* [10] and *Rnf8^−/−^* mice [30], [34], [35]. Rnf8 deficient males exhibit complete or partial infertility, impaired ubiquitylation of H2A in the XY body [34], and failure of global nucleosome removal; however, they are proficient in meiotic sex chromosome inactivation [35]. In contrast to *H2a.x^−/−^*, *Mdc1^−/−^* and *Rnf8^−/−^* males, *53bp1^−/−^* males are fertile, and 53bp1 is not required for meiotic recombination during spermatogenesis [28], [33]. While fertility of young *Rnf168^−/−^* males was normal, these males became infertile with age, and elderly mice displayed testicular degeneration and atrophy. These data indicate that Rnf168 plays important roles in spermatogenesis in an age-dependent manner.

Signaling of DSBs is critical for maintaining genomic integrity and suppressing cancer. Genomic instability and cancer susceptibility are increased in the absence of Mdc1, Rnf8, 53bp1 or Brca1 [10], [28], [30], [54]–[57]. Tumorigenesis in the absence of proteins involved in signaling of DSBs is further exacerbated in the absence of p53 as exemplified in *53bp1^−/−^p53^−/−^* mice [71]. While Celeste et al. reported that *H2a.x^−/−^* mice exhibit increased genomic instability, these mice did not show increased tumor susceptibility [27], [72]. However, inactivation of p53 strongly promoted tumorigenesis of *H2a.x^−/−^* mice [72], [73].

In this study, we demonstrate that Rnf168 deficiency also leads to increased radiosensitivity and genomic instability. However, similar to *H2a.x^−/−^* mice, tumor susceptibility was not increased in *Rnf168^−/−^* mice. We also demonstrate that Rnf168 and p53 collaborate in suppressing cancer since *Rnf168^−/−^p53^−/−^* mice exhibited shorter life spans and tumor latency compared to *p53^−/−^* littermates. These data indicate that p53 is critical for preventing tumorigenesis in *Rnf168^−/−^* mice. Therefore, not only is Rnf168 important for maintaining genomic stability, but it also collaborates with p53 to suppress cancer development.

While facial dysmorphism and short stature that are associated with RIDDLE syndrome were not observed in *Rnf168^−/−^* mice, these mutants displayed increased radiosensitivity, impaired CSR and immunodeficiency similar to the clinical features of this disease. Importantly, our data further highlight novel roles of Rnf168 in spermatogenesis, genomic integrity and cancer.

## Materials and Methods

### Generation of Rnf168-deficient mice

For the generation of Rnf168-deficient mice, two gene-trap ES clones, 156B6 and 405F11, were obtained from The Center for Modeling Human Disease (Toronto, Canada). In both ES clones, *Rnf168* gene was disrupted by the integration of the gene trap vectors between exons 5 and 6 of *Rnf168*. Both ES cell clones were successfully used to generate *Rnf168* mutant mice. Southern blotting and PCR analysis confirmed the disruption of *Rnf168* locus and were used to genotype the animals. The following primers were used for PCR genotyping: *Rnf168* mutant allele (forward 5′-ATCGCCTTCTATCGCCTTCT-3′ and reverse 5′-GCAGAAGACTCCGAACCTTG-3′), *Rnf168 WT* allele (forward 5′-GCCCAAGTCTGGCTCATTTA-3′ and reverse 5′-GCAGAAGACTCCGAACCTTG-3′). Southern blot analysis was performed using standard procedures. Rnf168 probe was generated by PCR using the following primers: 5′-TATTCCTGCTGCTGCTGCTA-3′, and 5′-CTCAAACCTCTTGCCCTCAG-3′. *Rnf168^−/−^* mice were generated by intercrossing *Rnf168^+/−^* mice obtained from each gene-trap clone. All mice in this study were in a mixed 129/J×C57BL/6 genetic background, and were maintained in a specific-pathogen free environment.

### Cell culture and generation of MEFs

MEFs were generated from day 12.5 embryos using standard procedures. MEFs were cultured in DMEM (Gibco Invitrogen Corporation) supplemented with 10% FCS. Splenocytes, thymocytes and lymphocytes were cultured under the same conditions in RPMI1640 (Gibco) with 10% FCS.

### Clonogenic assay

Mouse *Rnf168* cDNA amplified by PCR from *WT* MEF total cDNA was subcloned into pBABE-puro retroviral vector. Virus supernatant was collected 48 hour post transfection of phoenix cells with pBABE-puro-Rnf168. 3T3 immortalized MEFs were infected with Mock or Rnf168 retroviruses and were subjected to puromycin selection. Infected cells (1×10^3^) were seeded to 6 cm dishes, irradiated (2, 4 and 6 Gy) and cultured for 11 days. Number of colonies was counted with crystal violet staining.

### Flow cytometry

Single cell suspensions were stained with antibodies at 4°C in PBS with 1% FCS (Wincent, Inc.). The following antibodies conjugated to allophycocyanin, PE, FITC or perifinin chlorophyll protein were used for staining: anti-CD4, anti-CD8, anti-TCRβ, anti-TCRγδ, anti-Thy1.2, anti-B220, anti-CD25, anti-CD44, anti-IgG1, anti-IgG3 and anti-IgM (BD Bioscience, eBioscience). Stained cells were analyzed by FACS (FACSCalibur, BD Biosciences) using the CellQuest software (BD Biosciences) or FlowJo analysis software (Tree star).

### Cell death assay

LN cells (2×10^5^) were either treated with IR (0–4 Gy) or UV (0–80 J/m^2^). After 24 hours, cell death was examined using 7-aminoactinomycin D (Sigma).

### Cell cycle and checkpoints analysis

MEFs (passage 2–5) were synchronized using aphidicolin (Merck-Calbiochem) and either left untreated or treated with 10 Gy of IR. Subsequently, BrdU (Roche) was added to the cultures at various time points. MEFs were harvested, fixed using 70% ethanol and stained with FITC conjugated anti-BrdU (eBioscience) and PI. G2/M checkpoint was assessed by phospho-histone-H3 staining. Primary MEFs were either left untreated or treated with 2 Gy of IR. MEFs were harvested 1 hour post IR, fixed using 70% ethanol and stained with phospho-histone-H3 antibody (Cell signaling Technology) and PI.

### Western blot and immunoprecipitation analysis

Total protein extracts from cells were prepared using modified RIPA buffer (2 mM Tris-HCl (pH 7.5), 5 mM EDTA, 150 mM NaCl, 1% NP-40, 1% deoxycholate, 0.025% SDS, 1 mM phenylmethylsulfonyl fluoride and protease inhibitor cocktail tablets (Roche, Branchburg)). Proteins were separated on 10% homemade or 4–20% Tris-Glysine gradient polyacrylamide gels (Novex, Invitrogen). The following antibodies were used in 5% powdered milk (Carnation, Nestle) in TBS-T: affinity purified anti-Rnf168 antibodies (raised against either a murine GST-Rnf168^381–567^ and a murine Rnf168 N-terminal or against a murine GST-Rnf168), anti-Chk2 antibody (raised against murine Chk2^30–47^), anti-phospho Chk2 threonine 68 antibody (Cell Signaling Technology), anti-p53 antibody (FL393, SantaCruz), anti-phospho p53 serine 15 antibody (Cell Signaling Technology), anti-Nbs1 antibody (Novus Biologicals), anti-phospho Nbs1 serine 343 antibody (Novus Biologicals), anti-Smc1 antibody (Abcam), anti-phospho Smc1 (Abcam) and anti-Atm antibody (Cell Signaling Technology).

### Purification of splenic B-cells and *in vitro* CSR

B-cells were purified from spleen by negative selection using Dynal Mouse B-cell Negative Isolation kit (Dynal, Invitrogen). All B-cell experiments were performed using B-cells with a purity of 95.7±0.31%. B-cells (1×10^6^) were stimulated with mouse anti-CD40 antibody (10 µg/ml, eBioscience) or LPS (20 µg/ml, Sigma) plus recombinant mouse IL-4 (1000 U/ml, eBioscience) for 4 days, and switching of Ig to IgG1 and IgG2a isotypes was examined. LPS (15 µg/ml) was used to induce IgG2b and IgG3 switching.

### MSCV infection of primary B-cells

Mutants for *Rnf168* cDNA were generated by PCR. Wild-type and mutant *Rnf168* cDNAs were cloned into the MSCV-IRES-GFP vector. The ecotropic retroviruses expressing WT or mutated Rnf168 were generated by transient transfection of Phoenix cells with the appropriate retrovirus constructs. Purified splenic B-cells were activated with LPS and infected with virus supernatants containing polybrene (Sigma). Infected B-cells were cultured with LPS plus IL-4 for 4 days, and stained with anti-IgG1 for FACS analysis.

### Analysis of CSR by FACS

Purified B-cells were labeled with CFSE (Molecular Probes, Invitrogen) and cultured to induce IgG1 class switching for 4 days. Cells were stained with anti-IgG1 antibody and analyzed by FACS using the CellQuest software (BD Biosciences) or FlowJo analysis software (Tree star).

### Digestion-circularization PCR (DC-PCR)

Genomic DNA from B-cells stimulated with LPS plus IL-4 for 4 days was digested with EcoRI (New England Biolabs) overnight and ligated for 16 hour with T4 DNA ligase (New England Biolabs). Two rounds of PCR were performed using nested primer pairs for Sμ-Sγ1 and nAchR. Primer sequences for the first round of PCR are as follows: Sμ-Sγ1, 5′-GAGCAGCTACCAAGGATCAGGGA-3′ and 5′-CTTCACGCCACTGACTGACTGAG-3′; and AchR, 5′-GCAAACAGGGCTGGATGAGGCTG-3′ and 5′-GTCCCATACTTAGAACCCCAGCG-3′. Primer sequences for the second round of PCR are as follows: 5′-GGAGACCAATAATCAGAGGGAAG-3′ and 5′-GAGAGCAGGGTCTCCTGGGTAGG-3′; AchR, 5′-GGACTGCTGTGGGTTTCACCCAG-3′ and 5′-GCCTTGCTTGCTTAAGACCCTGG-3′.

### Analysis of Sμ-Sγ1 switch recombination junctions

Genomic DNA isolated from stimulated B-cells was amplified by PCR using *Pfu* ultra polymerase (Stratagene) and the following primers (5μ3, 5′-AATGGATACCTCAGTGGTTTTTAATGGTGGGTTTA-3′ and γ1-R, 5′-CAATTAGCTCCTGCTCTTCTGTGG-3′). PCR products (500–1000 bp) were cloned into pCR2.1 using the TOPO TA cloning kit (Invitrogen). Sequence analysis of the cloned PCR products was performed using Sequencher software (GeneCode) and NCBI-BLAST.

### ELISA

B-cells (1×10^6^) were stimulated with mouse anti-CD40 antibody or LPS (20 µg/ml) plus IL-4 (1000 U/ml) for 4 days and the levels of IgG1, IgG2a, and IgM isotypes in the culture supernatants were determined. Isotype switching to IgG2b and IgG3 was similarly examined using supernatants from B-cells stimulated with LPS (15 µg/ml). The levels of immunoglobulin in cell culture supernatants and serum from young (6–8 weeks) and old (9–12 months) mice were determined using SBA Clonotyping System/HRP and Mouse Immunoglobulin Isotype Panel (SouthernBiotech).

### Real-time PCR

Total RNA was isolated from MEFs using TRIzol reagent (Invitrogen) according to the manufacture's instructions. Total RNA (1 µg) was reverse transcribed using Superscript II (Invitrogen) and oligo(dT) primers according to the manufacturer's instructions. We performed RT-PCR using the following primers (Rnf168: forward 5′-GAATGTCAGTGCGGGATCTGTA-3′, reverse 5′-AGGGCTCTTCGTGTCACTCCTAT-3′, β-actin: forward 5′- TGTTACCAACTGGGACGACA-3′, reverse 5′-AAGGAAGGCTGGAAAAGAGC-3′).

Genomic DNA was extracted from total thymocytes and TCRδ rearrangement was assessed using the Applied Biosystems 7900HT Fast Real Time PCR System (Applied Biosystems), Power SYBR Green PCR Master Mix (Applied Biosystems) and specific primers as described previously [47].

### BrdU uptake experiments

*Rnf168^−/−^* and *WT* littermates received two intraperitoneal injections of BrdU (1 mg each) with 2 hours interval. Thymocytes collected from these mice, 2 hours after the second injection, were examined for BrdU incorporation using a BrdU Flow kit (Becton Dickinson) and cytofluorometry.

### Immunofluorescence microscopy

MEFs (passage 2–5) grown on glass slides were IR treated (5 Gy), and fixed with 2% paraformaldehide for 5 min at room temperature. Fixed MEFs were blocked with antibody dilution buffer (10% FCS and 0.05% Triton X-100 in PBS) and incubated with rabbit anti-53bp1 antibody (Bethyl Laboratories), rabbit anti-phospho-H2a.x (Ser139) antibody (Millipore), rabbit anti-Mdc1 antibody (Bethyl Laboratories), or a home made rabbit anti-Brca1 antibody (raised against a murine Brca1^831–845^) overnight at 4°C. Labeling was detected using Alexa Fluor 488-labeled goat-anti-rabbit immunoglobulin or Alexa Fluor 555-labeled goat-anti-mouse immunoglobulin secondary antibodies (Molecular Probes). Cells were stained with DAPI for 5 min and then mounted with Mowiol mount solution (Calbiochem). The slides were observed under a Leica DMIRB fluorescence microscope (Germany) equipped with digital camera (Leica DC 300RF). Images were acquired under 100× magnification using Leica Image Manager software. Foci-positive cells were quantified by manual counting.

Immortalized *Rnf168^−/−^* MEFs were either mock-transfected or transfected with wildtype RNF168 GFP-tagged expression vectors. 24 hours post transfection, cells were irradiated (5 Gy), fixed 1 hour later and stained with anti-53bp1 as described earlier.

Immortalized *WT* and *Rnf168^−/−^* MEFs were also treated with DMSO or DNA-PK inhibitors (NU7026 10 µM or NU7441 0.5 µM (TOCRIS)) prior to IR. Cells were irradiated (5 Gy), fixed 2 hours later, and stained with anti-γ-H2a.x as described earlier.

### Histology

Paraffin sections of testes, epididymides, tumors and organs were stained with hematoxylin-eosin for histological analyses. The slides were observed under a Leica DM4000B microscope (Germany) equipped with digital camera (Leica DC 300RF). Images were acquired using Leica Image Manager software.

### Cytogenetic analysis

Purified B-cells were activated with LPS for 48 hours, and either left untreated or irradiated as indicated. Cells were collected following 4 hours of colcemid treatment and processed by standard cytogenetic procedures. Number of chromosomes and gross chromosomal rearrangements were determined in 60 metaphase cells per sample from each genotype. The slides were observed under a Leica DMIRB fluorescence microscope (Germany) equipped with digital camera (Leica DC 300RF). Images were acquired under 100× magnification using Leica Image Manager software.

### Multicolor fluorescence in situ hybridization (mFISH)

Mouse mFISH probe kit was obtained from MetaSystems GmbH, Germany. In mFISH, all 21 chromosomes are each painted in a different color using combinatorial labeling. The stained metaphases were captured using the Axio Imager Z1 microscope (Carl Zeiss) with filter sets for FITC, Cy3.5, Texas Red, Cy5, Aqua, and DAPI. Images were captured, processed, and analyzed using ISIS mBAND/mFISH imaging software (MetaSystems). Detailed experimental procedures were outlined in earlier publications [74], [75].

### Statistical analysis

Data are presented as the mean ± SEM. The statistical significance of experimental data (p-values; Values≤0.05) was determined using the Wilcoxon test. Log-rank (Mantel-Cox) test was used for comparisons of survival curves.

### Ethics statement

All experiments were performed in compliance with Ontario Cancer Institute animal care committee guidelines.

## Supporting Information

Figure S1 Generation of *Rnf168* mutant mice. (A) Schematic representation of *wildtype* (*WT*) and mutants alleles of *Rnf168*. (B) Heterozygous and homozygous *Rnf168* mutant mice from 156B6 and 405F11 lines were identified by Southern blotting using EcoRV digested tail genomic DNA for 156B6, and EcoRV and KasI for 405F11 and 5′-flanking probe. Representative data from at least independent five experiments are shown. (C) Representative data of three independent RT-PCR experiments showing the expression levels of *Rnf168* transcripts in *WT* and *Rnf168^−/−^* MEFs from 156B6 and 405F11 lines. Actin is used as a control. (D, E) Expression of Rnf168 protein in MEFs and splenocytes from *WT* and *Rnf168^−/−^* mice. (D) MEF lysates were blotted with anti-Rnf168 antibody raised against C-terminal Rnf168 recombinant proteins. (E) Splenocyte whole cell lysates (WCL) were blotted with anti-Rnf168 antibody raised against recombinant full length Rnf168. Splenocyte lysates were IP using anti-Rnf168 antibody against the N-terminal Rnf168 and were blotted with anti-full length Rnf168 antibody. In *Rnf168^−/−^* splenocytes from 405F11 ES clone, gene trap construct derived YFP fused Rnf168 truncated proteins (1–256 amino acid) were detected. Representative data are shown from three independent experiments. * indicates non specific bands.
(0.44 MB TIF)

Figure S2 Cell cycle analysis of *WT* and *Rnf168^−/−^* MEFs. (A) Cell cycle analysis of aphidicolin synchronized *WT* and *Rnf168^−/−^* passage 2 MEFs. BrdU/PI assay and FACS analysis were used. Representative data are shown from three independent experiments.
(0.19 MB TIF)

Figure S3 Quantification of the effect of Rnf168 inactivation on IRIF for DDR proteins. (A) Quantitative analyses of 53bp1 nuclear foci are shown. *Rnf168^−/−^*, *Rnf8^−/−^* and *WT* MEFs were either untreated or exposed to 5 Gy of IR and fixed at the indicated times after IR. Three independent experiments were performed. (B) Quantitative analyses of the formation of Brca1 nuclear foci are shown. *Rnf168^−/−^* and *WT* MEFs were either untreated or exposed to 5 Gy of IR and were fixed at the indicated times after IR. Three independent experiments were performed. (C) Quantitative analyses of the formation of γ-H2a.x nuclear foci 6 hours post-IR. *Rnf168^−/−^* and *WT* MEFs were untreated or exposed to 5 Gy of IR. Three independent experiments were performed. (D) Quantitative analyses of the formation of Mdc1 nuclear foci. *Rnf168^−/−^* and *WT* MEFs were untreated or exposed to 5 Gy of IR and cells were fixed at the indicated times post-IR. Three independent experiments were performed. The data are presented as the mean ± SEM.
(0.20 MB TIF)

Figure S4 Effects of Rnf168 deficiency on the number of cells in lymphoid organs and on class switch recombination. (A) Absolute number of total, Pro-B (B220^+^IgM^−^CD43^+^) and Pre-B (B220^+^IgM^−^CD43^−^) BM cells from 6–8-week-old mice. Data are presented as the mean ± SEM. (n = 3). (B) Absolute numbers of splenocytes are shown. Data are presented as the mean ± SEM (n = 12–28). (C) Absolute numbers of LN cells are shown. Data are presented as the mean ± SEM (n = 12–28). (D) Representative two-color FACS analysis showing IgG1 expression on CFSE stained B-cells stimulated with LPS plus IL-4 for 4 days (left panels) and average percentages of IgG1 switched cells (right panel). Three independent experiments were performed. (E) CFSE staining profiles of *WT* and *Rnf168^−/−^* B-cells stimulated with LPS plus IL-4 for 4 days. (F) Expression levels of WT or mutated Rnf168 in B-cells infected with ecotropic retroviruses [MSCV-mutated or full-length (FL) Rnf168-IRES-GFP]. (G) Two independent DC-PCR experiments showing the effect of Rnf168 inactivation on Sμ-Sγ1 recombination. *nAchR* served to normalize for the amount of input DNA. Fivefold serial dilutions were used as templates. H_2_O: no input DNA.
(0.50 MB TIF)

Figure S5 Effect of Rnf168 deficiency on thymocytes. (A) Increased representation of CD4^−^CD8^−^ (DN) thymocytes in *Rnf168^−/−^* mice (n = 13) compared to *WT* controls (n = 14). 6–8-week-old mice were analyzed. Data are presented as the mean ± SEM. *p<0.05. (B) Reduced TCRγδ^+^ T-cells in *Rnf168^−/−^* (0.2±0.01%, n = 20) compared to *WT* controls (0.24±0.2%, n = 18). 6–8-week-old mice were analyzed. Data are presented as the mean ± SEM. *p<0.005. (C) Representative primary PCR data for genomic DNA rearrangements of Dδ1 to Dδ2, Dδ2 to Jδ1, and Vδ4 and Vδ5 to (D)Jδ1.
(0.27 MB TIF)

Figure S6 Tumors in *Rnf168^−/−^p53^−/−^* mice. (A and B) H&E staining of an hemangiosarcoma from an *Rnf168^−/−^p53^−/−^* mouse. (C and D) H&E staining of a sarcoma from an *Rnf168^−/−^p53^−/−^* mouse. (E and F) H&E staining of *Rnf168^−/−^p53^−/−^* thymoma invading lung (E) and salivary gland (F). (G and H) H&E staining showing *Rnf168^−/−^p53^−/−^* lymphoma cells invading lung (G) and liver (H). Scale Bars: 50 µm; (B), 100 µm; (D), 200 µm; (A, E, F, G and H), 500 µm; (C).
(2.70 MB TIF)

Table S1 Genotypes of pups from intercrosses of *Rnf168* heterozygotes. *Rnf168^−/−^* mice were viable and were born at the expected Mendelian ratio.
(0.03 MB DOC)

Table S2 Sequence analysis of Sμ-Sγ1 CSR junctions from *Rnf168^−/−^* and *WT* B-cells. In contrast to *WT* controls, a subset of CSR junctions in *Rnf168^−/−^* B-cells displays long nucleotide insertions.
(0.03 MB DOC)

Table S3 Distribution of tumors developed by *Rnf168^−/−^p53^−/−^* or *p53^−/−^* mice. *Rnf168^−/−^p53^−/−^* mice developed a different spectrum of tumors compared to *p53^−/−^* mice.
(0.05 MB DOC)

Table S4 mFISH Karyotype analysis of *Rnf168^−/−^p53^−/−^* tumors. Only clonal changes are shown and are described following ISCN 95 guidelines. (), numbers indicate chromosomes participating in cytogenetic aberrations. [], total number of cells exhibiting a particular chromosomal change are shown. dup, duplication of specified chromosomes. t, translocation.
(0.04 MB DOC)

## References

[1] Jackson SP, Bartek J (2009). The DNA-damage response in human biology and disease.. Nature.

[2] Zhou BB, Elledge SJ (2000). The DNA damage response: putting checkpoints in perspective.. Nature.

[3] Rouse J, Jackson SP (2002). Interfaces between the detection, signaling, and repair of DNA damage.. Science.

[4] Hakem R (2008). DNA-damage repair; the good, the bad, and the ugly.. Embo J.

[5] Bohgaki T, Bohgaki M, Hakem R (2010). DNA double-strand break signaling and human disorders.. Genome Integr.

[6] Stewart GS, Wang B, Bignell CR, Taylor AM, Elledge SJ (2003). MDC1 is a mediator of the mammalian DNA damage checkpoint.. Nature.

[7] Stucki M, Clapperton JA, Mohammad D, Yaffe MB, Smerdon SJ (2005). MDC1 directly binds phosphorylated histone H2AX to regulate cellular responses to DNA double-strand breaks.. Cell.

[8] Lee AC, Fernandez-Capetillo O, Pisupati V, Jackson SP, Nussenzweig A (2005). Specific association of mouse MDC1/NFBD1 with NBS1 at sites of DNA-damage.. Cell Cycle.

[9] Bekker-Jensen S, Lukas C, Kitagawa R, Melander F, Kastan MB (2006). Spatial organization of the mammalian genome surveillance machinery in response to DNA strand breaks.. The Journal of Cell Biology.

[10] Lou Z, Minter-Dykhouse K, Franco S, Gostissa M, Rivera MA (2006). MDC1 maintains genomic stability by participating in the amplification of ATM-dependent DNA damage signals.. Mol Cell.

[11] Goldberg M, Stucki M, Falck J, D'Amours D, Rahman D (2003). MDC1 is required for the intra-S-phase DNA damage checkpoint.. Nature.

[12] Spycher C, Miller ES, Townsend K, Pavic L, Morrice NA (2008). Constitutive phosphorylation of MDC1 physically links the MRE11-RAD50-NBS1 complex to damaged chromatin.. J Cell Biol.

[13] Melander F, Bekker-Jensen S, Falck J, Bartek J, Mailand N (2008). Phosphorylation of SDT repeats in the MDC1 N terminus triggers retention of NBS1 at the DNA damage-modified chromatin.. J Cell Biol.

[14] Wu L, Luo K, Lou Z, Chen J (2008). MDC1 regulates intra-S-phase checkpoint by targeting NBS1 to DNA double-strand breaks.. Proc Natl Acad Sci U S A.

[15] Chapman JR, Jackson SP (2008). Phospho-dependent interactions between NBS1 and MDC1 mediate chromatin retention of the MRN complex at sites of DNA damage.. EMBO Rep.

[16] Soutoglou E, Misteli T (2008). Activation of the cellular DNA damage response in the absence of DNA lesions.. Science.

[17] Kolas NK, Chapman JR, Nakada S, Ylanko J, Chahwan R (2007). Orchestration of the DNA-damage response by the RNF8 ubiquitin ligase.. Science.

[18] Mailand N, Bekker-Jensen S, Faustrup H, Melander F, Bartek J (2007). RNF8 ubiquitylates histones at DNA double-strand breaks and promotes assembly of repair proteins.. Cell.

[19] Huen MS, Grant R, Manke I, Minn K, Yu X (2007). RNF8 transduces the DNA-damage signal via histone ubiquitylation and checkpoint protein assembly.. Cell.

[20] Doil C, Mailand N, Bekker-Jensen S, Menard P, Larsen DH (2009). RNF168 binds and amplifies ubiquitin conjugates on damaged chromosomes to allow accumulation of repair proteins.. Cell.

[21] Pinato S, Scandiuzzi C, Arnaudo N, Citterio E, Gaudino G (2009). RNF168, a new RING finger, MIU-containing protein that modifies chromatin by ubiquitination of histones H2A and H2AX.. BMC Mol Biol.

[22] Stewart GS, Panier S, Townsend K, Al-Hakim AK, Kolas NK (2009). The RIDDLE syndrome protein mediates a ubiquitin-dependent signaling cascade at sites of DNA damage.. Cell.

[23] Huang J, Huen MS, Kim H, Leung CC, Glover JN (2009). RAD18 transmits DNA damage signalling to elicit homologous recombination repair.. Nat Cell Biol.

[24] Stewart GS, Stankovic T, Byrd PJ, Wechsler T, Miller ES (2007). RIDDLE immunodeficiency syndrome is linked to defects in 53BP1-mediated DNA damage signaling.. Proc Natl Acad Sci U S A.

[25] Lazzaro F, Giannattasio M, Puddu F, Granata M, Pellicioli A (2009). Checkpoint mechanisms at the intersection between DNA damage and repair.. DNA Repair (Amst).

[26] Xu B, Kim ST, Lim DS, Kastan MB (2002). Two molecularly distinct G(2)/M checkpoints are induced by ionizing irradiation.. Mol Cell Biol.

[27] Celeste A, Petersen S, Romanienko PJ, Fernandez-Capetillo O, Chen HT (2002). Genomic instability in mice lacking histone H2AX.. Science.

[28] Ward IM, Minn K, van Deursen J, Chen J (2003). p53 Binding protein 53BP1 is required for DNA damage responses and tumor suppression in mice.. Mol Cell Biol.

[29] Yuan J, Chen J (2009). MRE11/RAD50/NBS1 complex dictates DNA repair independent of H2AX.. J Biol Chem.

[30] Li L, Halaby MJ, Hakem A, Cardoso R, El Ghamrasni S (2010). Rnf8 deficiency impairs class switch recombination, spermatogenesis, and genomic integrity and predisposes for cancer.. J Exp Med.

[31] Stiff T, O'Driscoll M, Rief N, Iwabuchi K, Lobrich M (2004). ATM and DNA-PK function redundantly to phosphorylate H2AX after exposure to ionizing radiation.. Cancer Res.

[32] Fernandez-Capetillo O, Mahadevaiah SK, Celeste A, Romanienko PJ, Camerini-Otero RD (2003). H2AX is required for chromatin remodeling and inactivation of sex chromosomes in male mouse meiosis.. Dev Cell.

[33] Ahmed EA, van der Vaart A, Barten A, Kal HB, Chen J (2007). Differences in DNA double-strand breaks repair in male germ cell types: lessons learned from a differential expression of Mdc1 and 53BP1.. DNA Repair (Amst).

[34] Santos MA, Huen MS, Jankovic M, Chen HT, Lopez-Contreras AJ (2010). Class switching and meiotic defects in mice lacking the E3 ubiquitin ligase RNF8.. J Exp Med.

[35] Lu LY, Wu J, Ye L, Gavrilina GB, Saunders TL (2010). RNF8-dependent histone modifications regulate nucleosome removal during spermatogenesis.. Dev Cell.

[36] Jolly CJ, Cook AJ, Manis JP (2008). Fixing DNA breaks during class switch recombination.. J Exp Med.

[37] Stavnezer J, Guikema JE, Schrader CE (2008). Mechanism and regulation of class switch recombination.. Annu Rev Immunol.

[38] Hovelmeyer N, Wunderlich FT, Massoumi R, Jakobsen CG, Song J (2007). Regulation of B cell homeostasis and activation by the tumor suppressor gene CYLD.. J Exp Med.

[39] Lumsden JM, McCarty T, Petiniot LK, Shen R, Barlow C (2004). Immunoglobulin class switch recombination is impaired in Atm-deficient mice.. J Exp Med.

[40] Reina-San-Martin B, Chen J, Nussenzweig A, Nussenzweig MC (2007). Enhanced intra-switch region recombination during immunoglobulin class switch recombination in 53BP1−/− B cells.. Eur J Immunol.

[41] Yan CT, Boboila C, Souza EK, Franco S, Hickernell TR (2007). IgH class switching and translocations use a robust non-classical end-joining pathway.. Nature.

[42] Pan-Hammarstrom Q, Jones AM, Lahdesmaki A, Zhou W, Gatti RA (2005). Impact of DNA ligase IV on nonhomologous end joining pathways during class switch recombination in human cells.. J Exp Med.

[43] Reina-San-Martin B, Difilippantonio S, Hanitsch L, Masilamani RF, Nussenzweig A (2003). H2AX is required for recombination between immunoglobulin switch regions but not for intra-switch region recombination or somatic hypermutation.. J Exp Med.

[44] Reina-San-Martin B, Chen HT, Nussenzweig A, Nussenzweig MC (2004). ATM is required for efficient recombination between immunoglobulin switch regions.. J Exp Med.

[45] Manis JP, Morales JC, Xia Z, Kutok JL, Alt FW (2004). 53BP1 links DNA damage-response pathways to immunoglobulin heavy chain class-switch recombination.. Nat Immunol.

[46] Reina-San-Martin B, Nussenzweig MC, Nussenzweig A, Difilippantonio S (2005). Genomic instability, endoreduplication, and diminished Ig class-switch recombination in B cells lacking Nbs1.. Proc Natl Acad Sci U S A.

[47] Difilippantonio S, Gapud E, Wong N, Huang CY, Mahowald G (2008). 53BP1 facilitates long-range DNA end-joining during V(D)J recombination.. Nature.

[48] Morales JC, Xia Z, Lu T, Aldrich MB, Wang B (2003). Role for the BRCA1 C-terminal repeats (BRCT) protein 53BP1 in maintaining genomic stability.. J Biol Chem.

[49] Dudley EC, Petrie HT, Shah LM, Owen MJ, Hayday AC (1994). T cell receptor beta chain gene rearrangement and selection during thymocyte development in adult mice.. Immunity.

[50] Levelt CN, Eichmann K (1995). Receptors and signals in early thymic selection.. Immunity.

[51] von Boehmer H, Aifantis I, Feinberg J, Lechner O, Saint-Ruf C (1999). Pleiotropic changes controlled by the pre-T-cell receptor.. Curr Opin Immunol.

[52] Hoeijmakers JH (2009). DNA damage, aging, and cancer.. N Engl J Med.

[53] Lavin MF (2008). Ataxia-telangiectasia: from a rare disorder to a paradigm for cell signalling and cancer.. Nat Rev Mol Cell Biol.

[54] Xu X, Wagner KU, Larson D, Weaver Z, Li C (1999). Conditional mutation of Brca1 in mammary epithelial cells results in blunted ductal morphogenesis and tumour formation.. Nat Genet.

[55] Mak TW, Hakem A, McPherson JP, Shehabeldin A, Zablocki E (2000). Brcal required for T cell lineage development but not TCR loci rearrangement.. Nat Immunol.

[56] McPherson JP, Lemmers B, Hirao A, Hakem A, Abraham J (2004). Collaboration of Brca1 and Chk2 in tumorigenesis.. Genes Dev.

[57] Minter-Dykhouse K, Ward I, Huen MS, Chen J, Lou Z (2008). Distinct versus overlapping functions of MDC1 and 53BP1 in DNA damage response and tumorigenesis.. J Cell Biol.

[58] Donehower LA, Harvey M, Slagle BL, McArthur MJ, Montgomery CA (1992). Mice deficient for p53 are developmentally normal but susceptible to spontaneous tumours.. Nature.

[59] Jacks T, Remington L, Williams BO, Schmitt EM, Halachmi S (1994). Tumor spectrum analysis in p53-mutant mice.. Curr Biol.

[60] Donehower LA, Lozano G (2009). 20 years studying p53 functions in genetically engineered mice.. Nat Rev Cancer.

[61] Liao MJ, Zhang XX, Hill R, Gao J, Qumsiyeh MB (1998). No requirement for V(D)J recombination in p53-deficient thymic lymphoma.. Mol Cell Biol.

[62] Artandi SE, Chang S, Lee SL, Alson S, Gottlieb GJ (2000). Telomere dysfunction promotes non-reciprocal translocations and epithelial cancers in mice.. Nature.

[63] Celeste A, Fernandez-Capetillo O, Kruhlak MJ, Pilch DR, Staudt DW (2003). Histone H2AX phosphorylation is dispensable for the initial recognition of DNA breaks.. Nat Cell Biol.

[64] Lukas C, Melander F, Stucki M, Falck J, Bekker-Jensen S (2004). Mdc1 couples DNA double-strand break recognition by Nbs1 with its H2AX-dependent chromatin retention.. Embo J.

[65] Bekker-Jensen S, Lukas C, Melander F, Bartek J, Lukas J (2005). Dynamic assembly and sustained retention of 53BP1 at the sites of DNA damage are controlled by Mdc1/NFBD1.. The Journal of Cell Biology.

[66] Ramachandran S, Chahwan R, Nepal RM, Frieder D, Panier S (2010). The RNF8/RNF168 ubiquitin ligase cascade facilitates class switch recombination.. Proc Natl Acad Sci U S A.

[67] Ward IM, Reina-San-Martin B, Olaru A, Minn K, Tamada K (2004). 53BP1 is required for class switch recombination.. J Cell Biol.

[68] Dimitrova N, Chen YC, Spector DL, de Lange T (2008). 53BP1 promotes non-homologous end joining of telomeres by increasing chromatin mobility.. Nature.

[69] Bothmer A, Robbiani DF, Feldhahn N, Gazumyan A, Nussenzweig A (2010). 53BP1 regulates DNA resection and the choice between classical and alternative end joining during class switch recombination.. J Exp Med.

[70] Bunting SF, Callen E, Wong N, Chen HT, Polato F (2010). 53BP1 inhibits homologous recombination in Brca1-deficient cells by blocking resection of DNA breaks.. Cell.

[71] Ward IM, Difilippantonio S, Minn K, Mueller MD, Molina JR (2005). 53BP1 cooperates with p53 and functions as a haploinsufficient tumor suppressor in mice.. Mol Cell Biol.

[72] Celeste A, Difilippantonio S, Difilippantonio MJ, Fernandez-Capetillo O, Pilch DR (2003). H2AX haploinsufficiency modifies genomic stability and tumor susceptibility.. Cell.

[73] Bassing CH, Suh H, Ferguson DO, Chua KF, Manis J (2003). Histone H2AX: a dosage-dependent suppressor of oncogenic translocations and tumors.. Cell.

[74] Greulich KM, Kreja L, Heinze B, Rhein AP, Weier HG (2000). Rapid detection of radiation-induced chromosomal aberrations in lymphocytes and hematopoietic progenitor cells by mFISH.. Mutat Res.

[75] Hande MP, Azizova TV, Burak LE, Khokhryakov VF, Geard CR (2005). Complex chromosome aberrations persist in individuals many years after occupational exposure to densely ionizing radiation: an mFISH study.. Genes Chromosomes Cancer.

