# Supplementary figures and images for: Genomic Instability, Defective Spermatogenesis, Immunodeficiency, and Cancer in a Mouse Model of the RIDDLE Syndrome
Source: PLoS Genet. 2011 Apr 28;7(4):e1001381. doi: 10.1371/journal.pgen.1001381 (PMC3084200; doi:10.1371/journal.pgen.1001381)

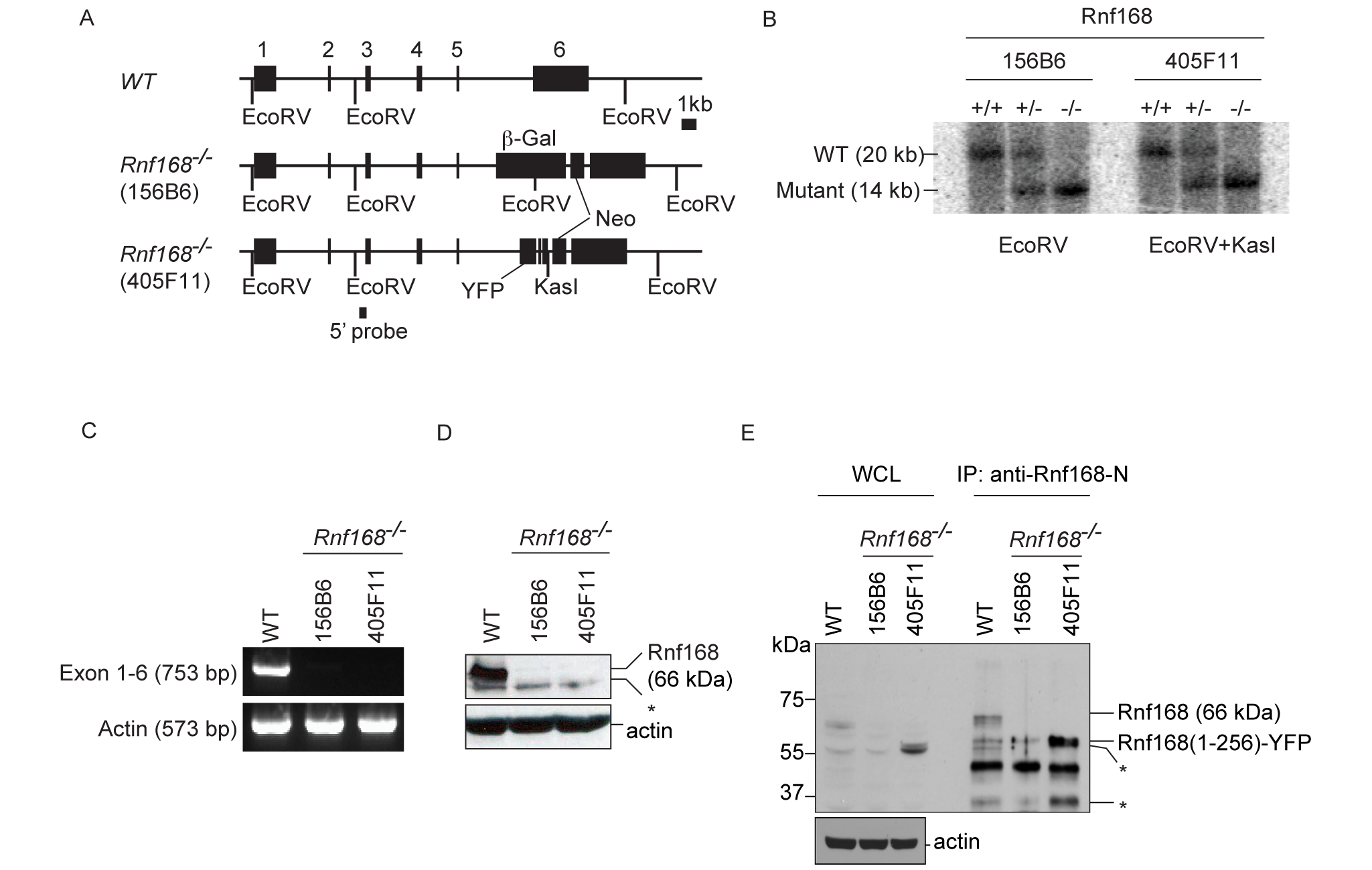

Supplement: Figure S1 — Generation of Rnf168 mutant mice. (A) Schematic representation of wildtype (WT) and mutants alleles of Rnf168. (B) Heterozygous and homozygous Rnf168 mutant mice from 156B6 and 405F11 lines were identified by Southern blotting using EcoRV digested tail genomic DNA for 156B6, and EcoRV and KasI for 405F11 and 5′-flanking probe. Representative data from at least independent five experiments are shown. (C) Representative data of three independent RT-PCR experiments showing the expression levels of Rnf168 transcripts in WT and Rnf168−/− MEFs from 156B6 and 405F11 lines. Actin is used as a control. (D, E) Expression of Rnf168 protein in MEFs and splenocytes from WT and Rnf168−/− mice. (D) MEF lysates were blotted with anti-Rnf168 antibody raised against C-terminal Rnf168 recombinant proteins. (E) Splenocyte whole cell lysates (WCL) were blotted with anti-Rnf168 antibody raised against recombinant full length Rnf168. Splenocyte lysates were IP using anti-Rnf168 antibody against the N-terminal Rnf168 and were blotted with anti-full length Rnf168 antibody. In Rnf168−/− splenocytes from 405F11 ES clone, gene trap construct derived YFP fused Rnf168 truncated proteins (1–256 amino acid) were detected. Representative data are shown from three independent experiments. * indicates non specific bands. (0.44 MB TIF) [file pgen.1001381.s001.tif]

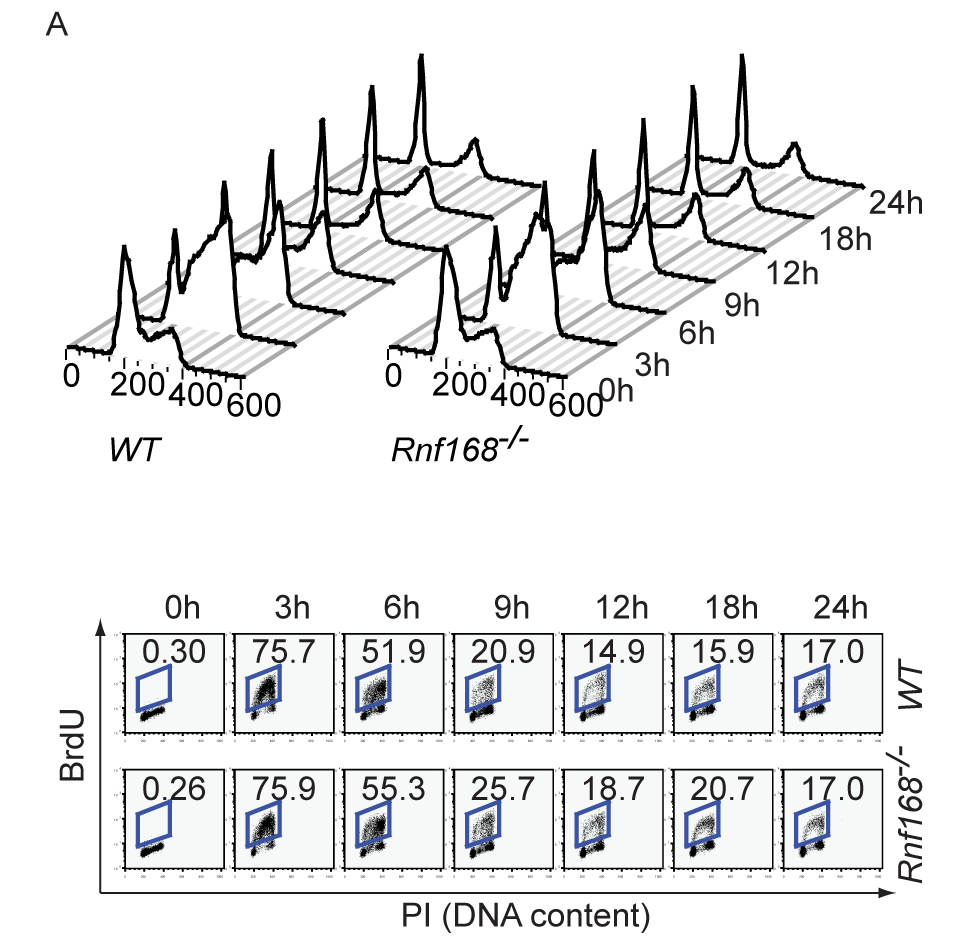

Supplement: Figure S2 — Cell cycle analysis of WT and Rnf168−/− MEFs. (A) Cell cycle analysis of aphidicolin synchronized WT and Rnf168−/− passage 2 MEFs. BrdU/PI assay and FACS analysis were used. Representative data are shown from three independent experiments. (0.19 MB TIF) [file pgen.1001381.s002.tif]

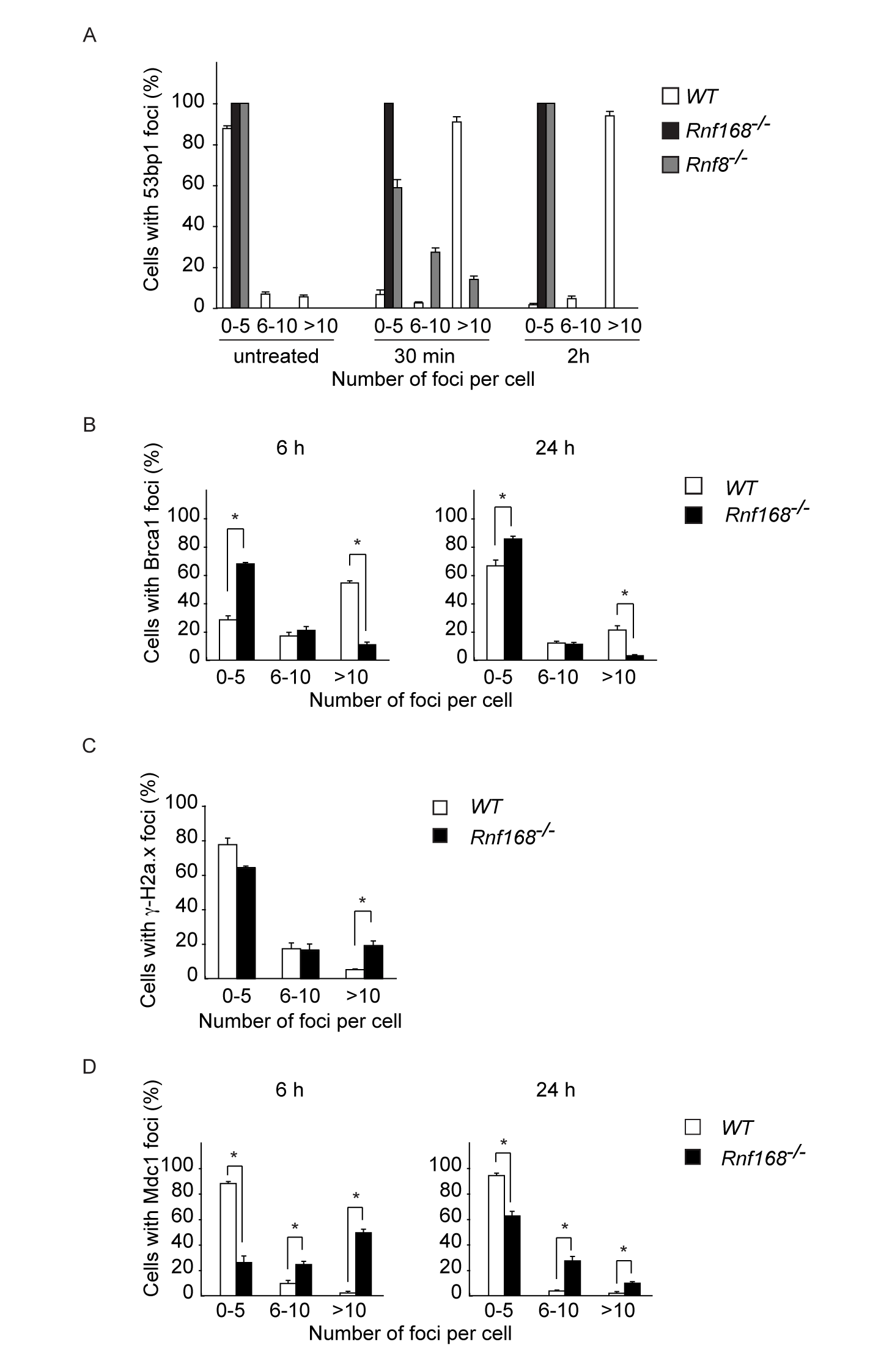

Supplement: Figure S3 — Quantification of the effect of Rnf168 inactivation on IRIF for DDR proteins. (A) Quantitative analyses of 53bp1 nuclear foci are shown. Rnf168−/−, Rnf8−/− and WT MEFs were either untreated or exposed to 5 Gy of IR and fixed at the indicated times after IR. Three independent experiments were performed. (B) Quantitative analyses of the formation of Brca1 nuclear foci are shown. Rnf168−/− and WT MEFs were either untreated or exposed to 5 Gy of IR and were fixed at the indicated times after IR. Three independent experiments were performed. (C) Quantitative analyses of the formation of γ-H2a.x nuclear foci 6 hours post-IR. Rnf168−/− and WT MEFs were untreated or exposed to 5 Gy of IR. Three independent experiments were performed. (D) Quantitative analyses of the formation of Mdc1 nuclear foci. Rnf168−/− and WT MEFs were untreated or exposed to 5 Gy of IR and cells were fixed at the indicated times post-IR. Three independent experiments were performed. The data are presented as the mean ± SEM. (0.20 MB TIF) [file pgen.1001381.s003.tif]

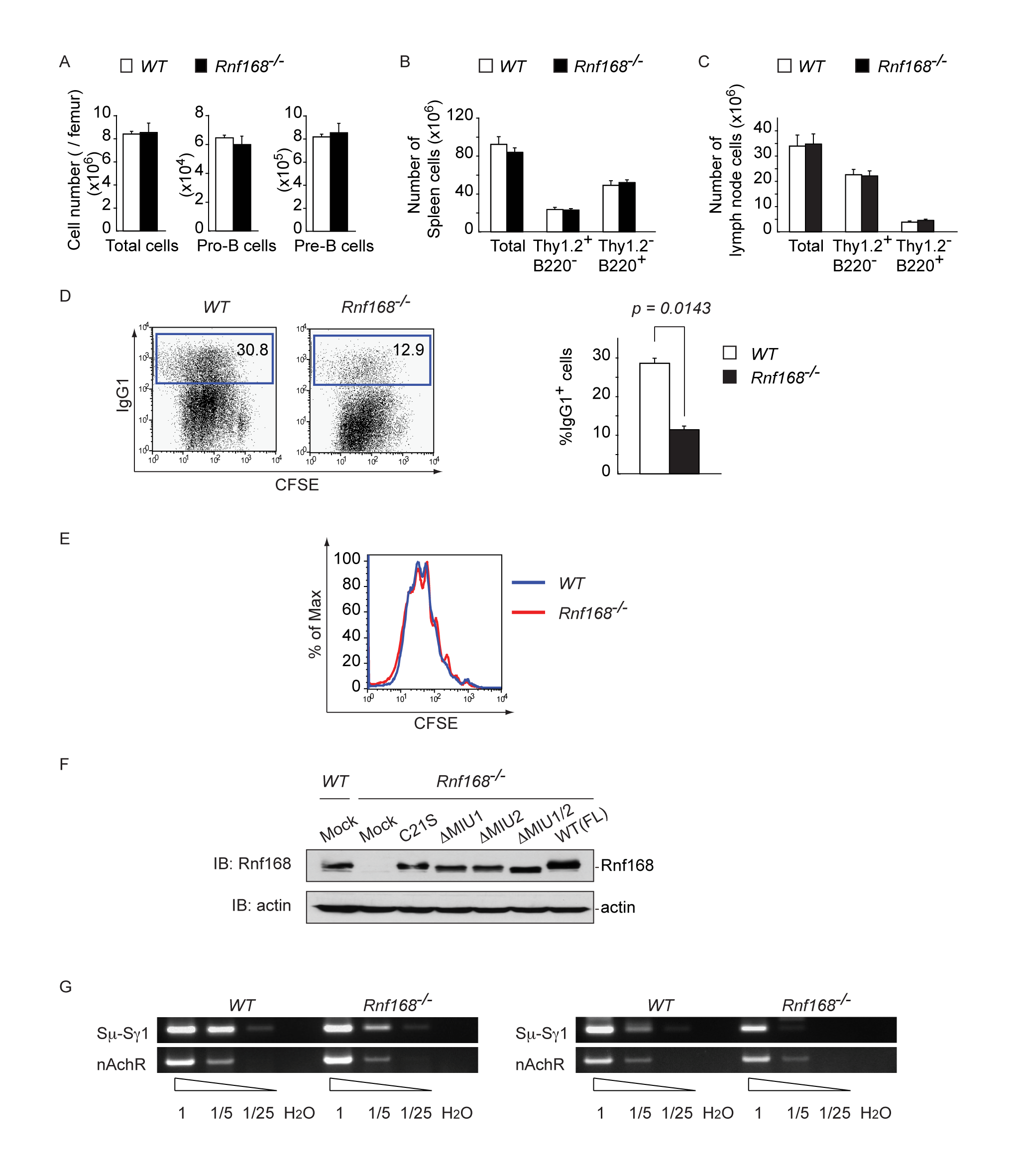

Supplement: Figure S4 — Effects of Rnf168 deficiency on the number of cells in lymphoid organs and on class switch recombination. (A) Absolute number of total, Pro-B (B220+IgM−CD43+) and Pre-B (B220+IgM−CD43−) BM cells from 6–8-week-old mice. Data are presented as the mean ± SEM. (n = 3). (B) Absolute numbers of splenocytes are shown. Data are presented as the mean ± SEM (n = 12–28). (C) Absolute numbers of LN cells are shown. Data are presented as the mean ± SEM (n = 12–28). (D) Representative two-color FACS analysis showing IgG1 expression on CFSE stained B-cells stimulated with LPS plus IL-4 for 4 days (left panels) and average percentages of IgG1 switched cells (right panel). Three independent experiments were performed. (E) CFSE staining profiles of WT and Rnf168−/− B-cells stimulated with LPS plus IL-4 for 4 days. (F) Expression levels of WT or mutated Rnf168 in B-cells infected with ecotropic retroviruses [MSCV-mutated or full-length (FL) Rnf168-IRES-GFP]. (G) Two independent DC-PCR experiments showing the effect of Rnf168 inactivation on Sμ-Sγ1 recombination. nAchR served to normalize for the amount of input DNA. Fivefold serial dilutions were used as templates. H2O: no input DNA. (0.50 MB TIF) [file pgen.1001381.s004.tif]

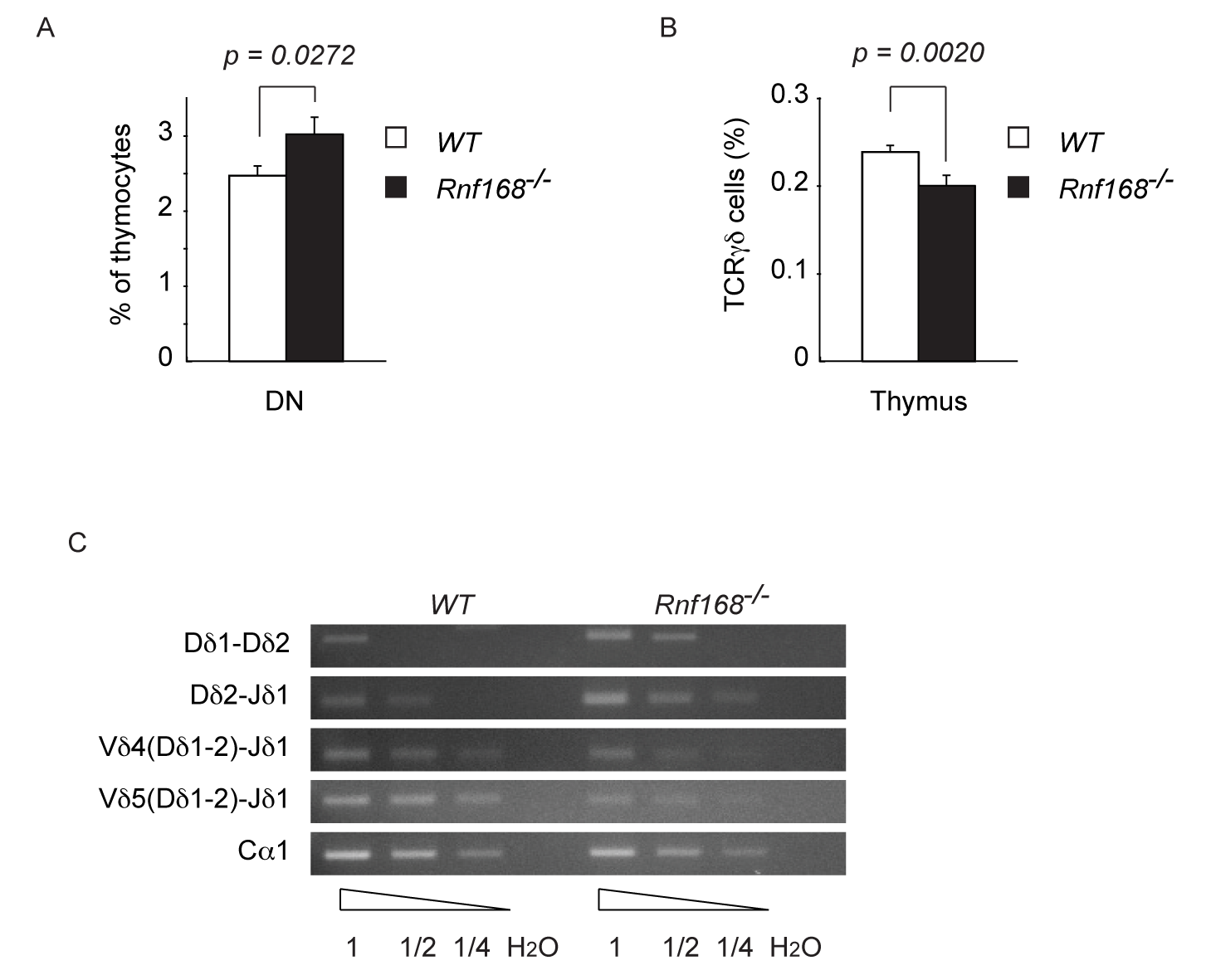

Supplement: Figure S5 — Effect of Rnf168 deficiency on thymocytes. (A) Increased representation of CD4−CD8− (DN) thymocytes in Rnf168−/− mice (n = 13) compared to WT controls (n = 14). 6–8-week-old mice were analyzed. Data are presented as the mean ± SEM. *p<0.05. (B) Reduced TCRγδ+ T-cells in Rnf168−/− (0.2±0.01%, n = 20) compared to WT controls (0.24±0.2%, n = 18). 6–8-week-old mice were analyzed. Data are presented as the mean ± SEM. *p<0.005. (C) Representative primary PCR data for genomic DNA rearrangements of Dδ1 to Dδ2, Dδ2 to Jδ1, and Vδ4 and Vδ5 to (D)Jδ1. (0.27 MB TIF) [file pgen.1001381.s005.tif]

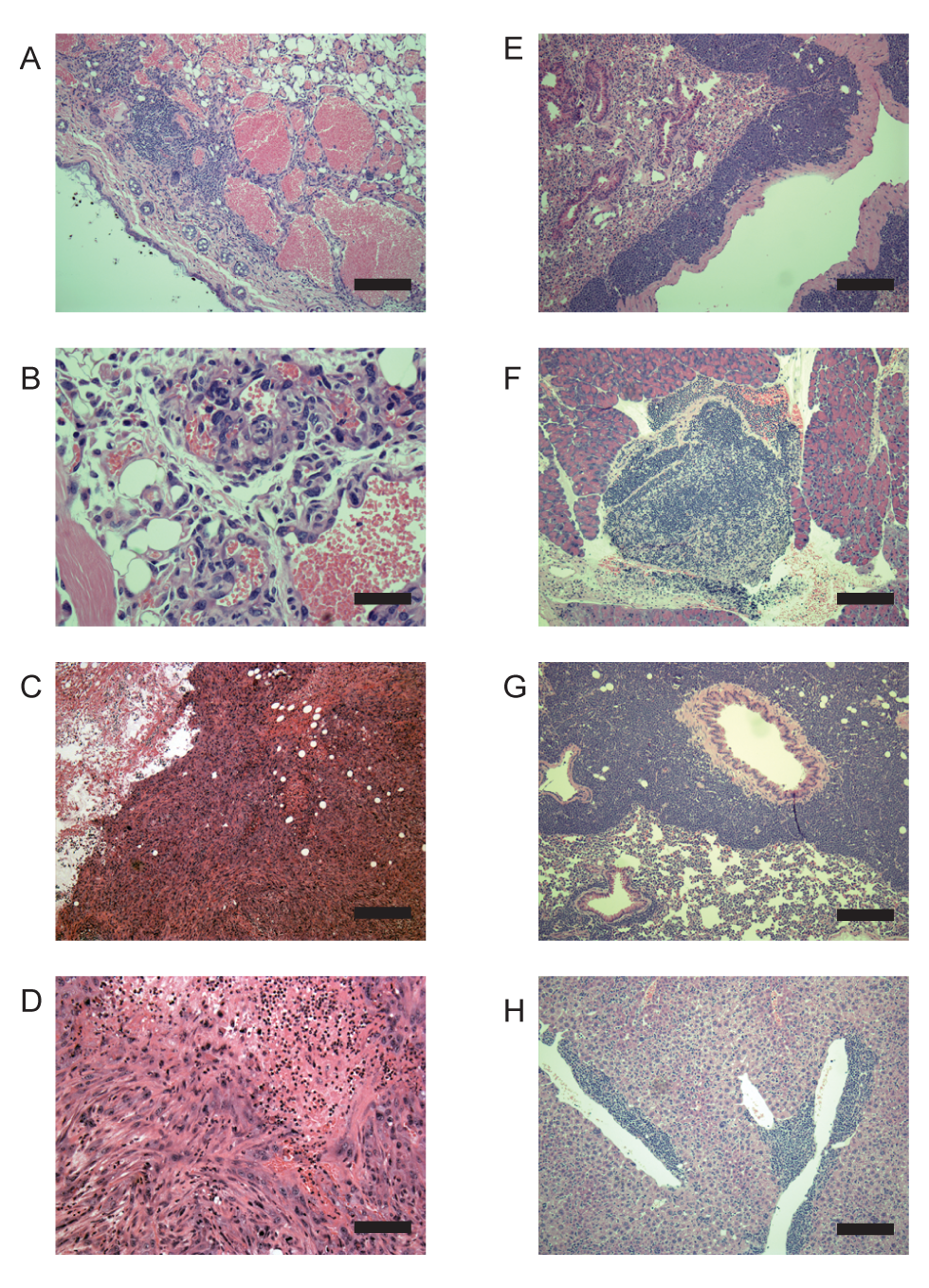

Supplement: Figure S6 — Tumors in Rnf168−/−p53−/− mice. (A and B) H&E staining of an hemangiosarcoma from an Rnf168−/−p53−/− mouse. (C and D) H&E staining of a sarcoma from an Rnf168−/−p53−/− mouse. (E and F) H&E staining of Rnf168−/−p53−/− thymoma invading lung (E) and salivary gland (F). (G and H) H&E staining showing Rnf168−/−p53−/− lymphoma cells invading lung (G) and liver (H). Scale Bars: 50 µm; (B), 100 µm; (D), 200 µm; (A, E, F, G and H), 500 µm; (C). (2.70 MB TIF) [file pgen.1001381.s006.tif]
