# Supplementary material for: Genomic Instability, Defective Spermatogenesis, Immunodeficiency, and Cancer in a Mouse Model of the RIDDLE Syndrome
Source: PLoS Genet. 2011 Apr 28;7(4):e1001381. doi: 10.1371/journal.pgen.1001381 (PMC3084200; doi:10.1371/journal.pgen.1001381)
Supplement: Table S1 — Genotypes of pups from intercrosses of Rnf168 heterozygotes. Rnf168−/− mice were viable and were born at the expected Mendelian ratio. (0.03 MB DOC) [file pgen.1001381.s007.doc]

**Table S1. Genotypes of pups from intercrosses of *Rnf168* heterozygotes**

| *Rnf168^+/-^* Strain | Genotype | | |
| --- | --- | --- | --- |
|  | +/+ | +/- | -/- |
| 156B6 | 73 | 158 | 76 |
| 405F11 | 93 | 171 | 88 |

*Rnf168^-/-^* mice were viable and were born at the expected Mendelian ratio.
