# Supplementary material for: Genomic Instability, Defective Spermatogenesis, Immunodeficiency, and Cancer in a Mouse Model of the RIDDLE Syndrome
Source: PLoS Genet. 2011 Apr 28;7(4):e1001381. doi: 10.1371/journal.pgen.1001381 (PMC3084200; doi:10.1371/journal.pgen.1001381)
Supplement: Table S2 — Sequence analysis of Sμ-Sγ1 CSR junctions from Rnf168−/− and WT B-cells. In contrast to WT controls, a subset of CSR junctions in Rnf168−/− B-cells displays long nucleotide insertions. (0.03 MB DOC) [file pgen.1001381.s008.doc]

**Table S2. Sequence analysis of Sμ-Sγ1 CSR junctions from *Rnf168^-/-^* and *WT* B-cells**

|  | Nucleotide overlap (bp) | Junctions with insertions (%) | Sequences analyzed |
| --- | --- | --- | --- |
| *WT* | 1.19 ± 0.24 | 0.0 | 37 |
| *Rnf168^-/-^* | 1.03 ± 0.22 | 5.1 | 39 |

In contrast to *WT* controls, a subset of CSR junctions in *Rnf168^-/-^* B-cells displays long nucleotide insertions.
