# Supplementary material for: Genomic Instability, Defective Spermatogenesis, Immunodeficiency, and Cancer in a Mouse Model of the RIDDLE Syndrome
Source: PLoS Genet. 2011 Apr 28;7(4):e1001381. doi: 10.1371/journal.pgen.1001381 (PMC3084200; doi:10.1371/journal.pgen.1001381)
Supplement: Table S3 — Distribution of tumors developed by Rnf168−/−p53−/− or p53−/− mice. Rnf168−/−p53−/− mice developed a different spectrum of tumors compared to p53−/− mice. (0.05 MB DOC) [file pgen.1001381.s009.doc]

**Table S3. Distribution of tumors developed by *Rnf168^-/-^p53^-/-^* or *p53^-/-^*** mice.

| Mouse ID | Rnf168  ES clone | Genotype | Tumor type |
| --- | --- | --- | --- |
| p53RF4-4 | 405F11 | *Rnf168^-/-^p53^-/-^* | Lymphoma |
| p53RF7-2 | 405F11 | *Rnf168^-/-^p53^-/-^* | Thymoma, Hemangiosarcoma |
| p53RF11-8 | 405F11 | *Rnf168^-/-^p53^-/-^* | B-cell lymphoma |
| p53RB7-2 | 156B6 | *Rnf168^-/-^p53^-/-^* | B-cell lymphoma |
| p53RB13-3 | 156B6 | *Rnf168^-/-^p53^-/-^* | Sarcoma |
| p53RB14-6 | 156B6 | *Rnf168^-/-^p53^-/-^* | B-cell lymphoma |
| p53RB19-9 | 156B6 | *Rnf168^-/-^p53^-/-^* | B-cell lymphoma |
| p53RB20-4 | 156B6 | *Rnf168^-/-^p53^-/-^* | Colon tumor, Hemorrhagic ascites |
| p53RB20-5 | 156B6 | *Rnf168^-/-^p53^-/-^* | Thymoma |
| p53RB20-6 | 156B6 | *Rnf168^-/-^p53^-/-^* | Thymoma |
| p53RB31-2 | 156B6 | *Rnf168^-/-^p53^-/-^* | Testicular tumor |
|  |  |  |  |
| p53RF10-7 |  | *p53^-/-^* | Thymoma |
| p53RB6-2 |  | *p53^-/-^* | Thymoma |
| p53RB8-7 |  | *p53^-/-^* | Thymoma |
| p53RB9-2 |  | *p53^-/-^* | Thymoma |
| p53RB11-9 |  | *p53^-/-^* | Thymoma |
| p53RB18-2 |  | *p53^-/-^* | Thymoma |

*Rnf168^-/-^p53^-/-^* mice developed a different spectrum of tumors compared to *p53^-/-^* mice.
