# Supplementary material for: Genomic Instability, Defective Spermatogenesis, Immunodeficiency, and Cancer in a Mouse Model of the RIDDLE Syndrome
Source: PLoS Genet. 2011 Apr 28;7(4):e1001381. doi: 10.1371/journal.pgen.1001381 (PMC3084200; doi:10.1371/journal.pgen.1001381)
Supplement: Table S4 — mFISH Karyotype analysis of Rnf168−/−p53−/− tumors. Only clonal changes are shown and are described following ISCN 95 guidelines. (), numbers indicate chromosomes participating in cytogenetic aberrations. [], total number of cells exhibiting a particular chromosomal change are shown. dup, duplication of specified chromosomes. t, translocation. (0.04 MB DOC) [file pgen.1001381.s010.doc]

**Table S4. mFISH Karyotype analysis of *Rnf168^-/-^p53^-/-^* tumors**.

| **Tumor**  **type** | B-cell  lymphoma | B-cell  lymphoma | B-cell  lymphoma | Thymoma |
| --- | --- | --- | --- | --- |
| **Mouse ID** | p53RB7-2 | p53RB14-6 | p53RF11-8 | p53RF7-2 |
| **Karyotype** | 4 x 40, XY  4 x 39, XY  2 x 38, XY  t(12;15), t(15;12) [10]  t(9;11) [6]  del(9) [3] | 3 x 40, XY  2 x 39, XY  t(12;15),  t(15;12) [4] | 5 x 39~42, XY  del(4) [4] | 6 x 42~54, XY  dup(2) [3], dup(4) [5] dup(5) [5], dup(7) [4] dup(11) [6], dup(14) [4] dup(15) [6] |

Only clonal changes are shown and are described following ISCN 95 guidelines.

(), numbers indicate chromosomes participating in cytogenetic aberrations. [], total number of cells exhibiting a particular chromosomal change are shown. dup, duplication of specified chromosomes. t, translocation.
